# Estimating seepage in heterogeneous earthfill dams on permeable foundations using explainable machine learning

**DOI:** 10.1038/s41598-026-45048-5

**Published:** 2026-04-10

**Authors:** Mohamed Maher Sayed Ahmed, Muhammad Zeeshan Khursheed, Badee Alshameri, Abdelrahman Kamal Hamed, Mohamed Kamel Elshaarawy

**Affiliations:** 1 Civil Engineering Department, Faculty of Engineering, Horus University-Egypt, New Damietta, 34517 Egypt; 2 National University of Sciences and Technology, Islamabad, Pakistan; 3 Irrigation and Hydraulics Engineering Department, Faculty of Engineering, Tanta University, Tanta, 31733 Egypt; 4 Key Laboratory of Ministry of Education for Geomechanics and Embankment Engineering, Hohai University, Nanjing, China

**Keywords:** Non-homogenous earthen dams, Permeable foundation, Seepage Discharge, Machine learning, SHAP, Engineering, Environmental sciences, Hydrology, Natural hazards

## Abstract

Predicting seepage discharge through heterogeneous earthfill dams founded on permeable foundations is essential for dam safety and long-term water-resources sustainability. Accordingly, this study evaluated five machine learning (ML) models viz Decision Tree (DT), Random Forest (RF), Stochastic Gradient Boosting (SGB), Light Gradient Boosting (LGB), and Categorical Gradient Boosting (CGB), to estimate seepage discharge using seven geometric and hydraulic input variables. The dataset was partitioned into training (80%), validation (10%), and testing (10%) subsets. To improve predictive capability, Bayesian Optimization (BO) was applied for hyperparameter tuning. Subsequently, model performance was examined using multiple error metrics, predicted–actual scatter plots, SHapley Additive exPlanations (SHAP) for interpretability, and k-fold cross-validation; finally, a rank-based analysis was used to consolidate the results across evaluation criteria. Overall, the tuned models exhibited substantial performance gains. In particular, the boosting-based methods consistently outperformed the DT and RF baselines. Among all candidates, the CGB model achieved the best overall performance, delivering near-perfect accuracy (R² = 0.9981 on validation and R² = 0.996 on testing). Moreover, cross-validation confirmed its robustness, as it produced the lowest RMSE values across the 10 folds. SHAP-based analysis further indicated that reservoir water depth and the core-to-shell hydraulic conductivity ratio are the dominant drivers of seepage behavior, whereas dam crest width and foundation depth exert secondary influence. To support practical use, a standalone desktop GUI was also developed to enable instant predictions with flexible input formats, batch evaluation, and reproducible export functions. Collectively, these results demonstrate that the CGB model provides a reliable and deployable framework for seepage prediction, maintaining prediction differences below 10% relative to prior numerical and empirical references across varying water depths.

## Introduction

Dams function as critical water-retention infrastructure, engineered to achieve multiple purposes, including water supply for domestic and agricultural use, renewable energy generation, flood control, and navigation support^1^. The structural integrity essential for these functions is significantly dependent on controlling water seepage in earthfill dams. When seepage exceeds safe limits, it can initiate internal erosion, a process known as piping, which progressively enlarges internal flow paths and increases soil permeability, ultimately threatening the stability of the entire dam^2^. The critical nature of this failure mechanism is highlighted by Fell et al.^3^, which attributes a significant quarter of all earthfill dam failures directly to seepage-related issues.

Consequently, accurately predicting the quantity of seepage flow, known as seepage discharge, has become a primary focus in the design and safety assessment of these dams. The goal of identifying reliable methods for quantifying seepage has been investigated over a century ago^4–6^. Subsequently, Rozanov^7^ and Stello^8^ developed further analytical solutions to refine seepage analysis. This evolution became evident in modern standardized guidelines, such as those published by the United States Bureau of Reclamation^9^. Despite their historical value, these analytical methods are inherently constrained by simplifying assumptions that restrict their application only to dams with relatively simple and uniform geometries.

To overcome these limitations, the Finite Element Method (FEM) has emerged as a powerful computational tool capable of modeling complex seepage scenarios^10^. Its early adoption in dam engineering is evidenced by previous studies by Hillo^11^ and Dunbar and Sheahan^12^. Khsaf^13^ and Irzooki^14^ further demonstrated its versatility by modeling seepage through structures with flow control devices and in earthfill dams, respectively. Recently, FEM has remained the predominant methodology for seepage analysis. Al-Damluji et al.^15^ combined FEM with boundary element methods for steady-state conditions, while Kamanbedast and Delvari^16^ applied commercial FEM software like GeoStudio to model the Maroon dam. Olonade and Agbede^17^ evaluated seepage discharge from the Oba dam. Komasi and Beiranvand^18^ studied the seepage and stability of Eyvashan dam under rapid drawdown. Furthermore, Salmasi and Abraham^19^ systematically validated the superiority of the FEM by quantitatively comparing its results against traditional analytical approaches.

One of the most trusted programs is SEEP/W (part of the GeoStudio package), which is used by engineers worldwide to accurately model water flow and ensure the safety and longevity of dams and was profoundly applicable based on earlier studies^20–22^. Subsequent research has further solidified its utility by applying SEEP/W to a wide range of specialized scenarios. These applications include analyzing zoned earth dams^23^, modeling dams on permeable foundations^24^, evaluating the performance of cutoff walls^25^, simulating both steady-state and transient seepage conditions^26^, investigating the role of clay cores^27^, and refining the estimation of seepage discharge^28^.

More recently, the field has witnessed a paradigm shift with the integration of Artificial Intelligence (AI) and machine learning (ML) for seepage and pore water pressure modeling. Artificial Neural Networks (ANNs) have been at the forefront of this advancement. Tayfur et al.^29^ demonstrated the potential of ANNs by showing their superiority over FEM in estimating seepage for the Jeziorsko earthfill dam. Baghalian et al.^30^ and Bhattacharjya and Sen^31^ further developed ANN models for predicting seepage through dam foundations and homogeneous earthfill dams, consistently reporting high predictive accuracy. The effectiveness of ANNs continues to be validated in Rehamnia et al.^32^, achieving exceptional performance for dams with cores. Beyond standalone ANNs, the exploration of hybrid AI models and other ML techniques has expanded the toolbox available to engineers. Beiranvand and Rajaee^33^ of 46 studies highlighted the efficacy of hybrid models as well as single models like Support Vector Regression (SVR) and Random Forest (RF). Back Propagation Neural Networks (BPNN) and a BPNN-Genetic Algorithm (GA) hybrid were employed to predict piezometric levels, underscoring the growing advancement and potential of data-driven approaches in dam safety analysis^34^.

Ziggah et al.^35^ investigated the use of AI and ML models, including BPNN, group method of data handling (GMDH), radial basis function neural network (RBFNN), least squares support vector machine (LSSVM), support vector machine (SVM), M5 prime (M5P), and Gaussian process regression (GPR) for predicting piezometric water levels in dam seepage analysis. The results indicated that the GMDH model consistently outperformed the others. Pandey et al.^36^ demonstrated a hybrid GA-ANN model, developed using long-term groundwater recharge and discharge. They found that the GA-ANN significantly outperformed traditional GA models in predicting seasonal groundwater table depth. Zhang et al.^37^ used hybrid AI-based models (i.e., GA-BPNN), which outperform conventional statistical approaches in dam seepage prediction by effectively capturing nonlinear relationships between seepage behavior and loading conditions. VaeziNejad et al.^38^ proposed a hybrid intelligent inverse modeling approach that integrates an FEM transient seepage model with BPNN and Particle Swarm Optimization (PSO) to identify leakage sources in earth dams, demonstrating high accuracy and robustness when applied to the Baft Dam case study. Khorchani et al.^39^ showed soft computing techniques, including feed-forward neural networks (FFNN), RBFNN, and SVM, for predicting piezometric levels of seepage behavior in earthfill dams. Comparative results showed that the FFNN model achieved superior accuracy over RBF and SVM models.

## Research gap

Despite advancements in finite element–based and empirical approaches for predicting seepage in earthfill dams, significant gaps remain in modeling non-homogeneous dam structures founded on permeable layers. Existing studies, such as Suleimany and Mamand^40^, primarily focus on homogeneous dams or neglect the coupled effects of material heterogeneity and foundation permeability, which govern complex seepage paths in real dam systems. Moreover, although analytical and numerical simulations have been extensively employed, the application of ML techniques for seepage prediction remains limited, with most available ML-based studies relying on restricted, site-specific, or numerically generated datasets and simplified foundation assumptions. Consequently, current ML models do not adequately capture the combined influence of heterogeneous dam zoning and permeable foundations, highlighting the need for a more generalizable and physically consistent ML framework. Thus, the main contributions of the present study can be summarized as follows:

- To quantify the joint effect of geometric and hydraulic parameters for the dam body and foundation on seepage discharge using a curated database of 4374 numerical scenarios.
- To perform descriptive and correlation analyses to characterize variability, dominant trends, and potential multicollinearity among inputs.
- To develop and benchmark five ML models: DT, RF, SGB, LGB, and CGB tailored to nonlinear, tabular regression of seepage discharge.
- To optimize model hyperparameters using Bayesian Optimization with five-fold cross-validation, followed by ten-fold cross-validation to enhance generalization and control overfitting.
- To evaluate predictive performance using a multi-metric suite augmented by REC curves, scatter plots, and rank aggregation strategy.
- To enhance interpretability through SHAP, quantifying global and local feature effects and revealing interactions that drive seepage discharge.
- To operationalize the best-performing model via an offline GUI that accepts inputs and returns immediate predictions for practitioner-oriented scenario analysis and decision support.

## Materials and methods

**Fig. 1:**
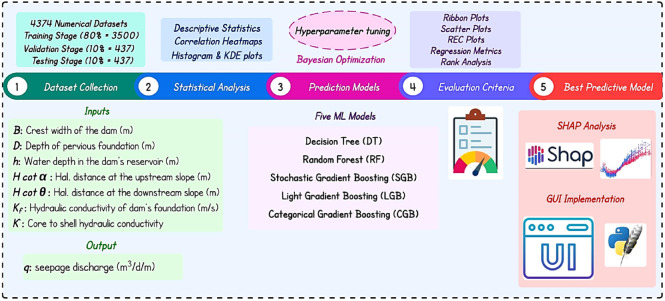
Flowchart of research methodology.

Figure 1 outlines the end-to-end workflow for modeling seepage discharge through a non-homogeneous earthfill dam resting on a permeable foundation. A comprehensive database comprising 4,374 numerically simulated scenarios is generated to represent a wide range of hydraulic, geometric, and geotechnical conditions. The dataset is randomly split into 80% for training, 10% for validation, and 10% for testing, enabling robust hyperparameter optimization and independent evaluation of model generalization. The original variables was reformulated into seven physically meaningful input parameters, while seepage discharge per unit dam length (*q*) is considered as the model output. An initial statistical characterization of the dataset is performed using descriptive statistics to evaluate data dispersion and distribution. Five supervised ML regression models are investigated: DT, RF, SGB, LGB, and CGB. Model development is conducted within a BO framework, systematically tuning key hyperparameters to achieve an optimal balance between predictive accuracy and model generalization.

Model performance is evaluated using standard regression metrics and further scrutinized through REC curves, ribbon plots, and observed–predicted scatter plots, allowing both global and local performance assessment. To objectively identify the most reliable predictor, a rank aggregation strategy was applied across all evaluation criteria. The SHAP analysis is employed to quantify feature contributions and reveal interaction effects governing seepage behavior. Finally, the optimal model was embedded within a desktop-based GUI, enabling real-time seepage prediction and facilitating scenario-based analysis for practical engineering applications.

### Database collection

Numerical modeling is a robust and widely accepted approach for analyzing seepage behavior in earthfill dams. In the present study, the seepage database and the corresponding numerical modeling framework were collected from the methodology reported by Khursheed et al.^41^, which was implemented using the GeoStudio software package (SEEP/W module) developed by GEO-SLOPE International^42^. The adopted SEEP/W-based methodology simulates steady-state seepage through earthfill dams and internal diaphragm walls, enabling evaluation of seepage flow paths, pore-water pressure distributions, and seepage discharge per unit dam length.

**Fig. 2:**
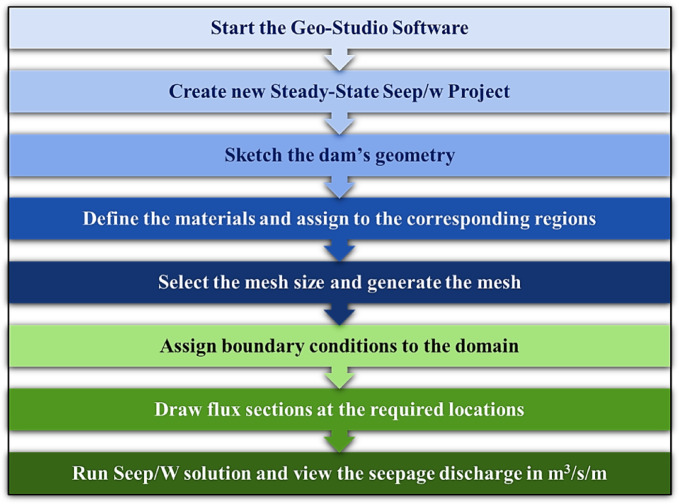
Methodological steps of the SEEP/W model to generate the collected database^41^.

The numerical modeling workflow used to generate the database follows the procedure described by Khursheed et al.^41^ and is schematically illustrated in Fig. 2. In brief, the workflow includes: (i) initializing a steady-state SEEP/W project and defining the dam geometry; (ii) assigning hydraulic and geotechnical properties to the corresponding material zones; (iii) generating the finite-element mesh with appropriate element sizing to ensure numerical stability and solution accuracy; (iv) prescribing upstream and downstream hydraulic boundary conditions; (v) defining flux sections at selected locations to quantify seepage discharge; and (vi) executing the SEEP/W solver to compute seepage discharge per unit dam length (m³/s/m). Using this adopted methodology, a comprehensive database comprising 4,374 numerically simulated scenarios was collected from Khursheed et al.^41^ to represent a wide range of hydraulic, geometric, and geotechnical conditions. This dataset was subsequently used as the basis for developing and evaluating the proposed ML models for seepage discharge prediction.

#### Governing equations

The seepage analysis in SEEP/W is based on Darcy’s law, which describes fluid flow through porous media under both saturated and unsaturated conditions. Darcy’s law links the seepage discharge to the hydraulic conductivity, hydraulic gradient, and cross-sectional flow area, as shown in Eq. (1):

1 $$\:Q=KIA$$

where *Q* is the seepage discharge, *K* is the hydraulic conductivity, *I* is the hydraulic gradient, and *A* is the cross-sectional area normal to the flow direction. Under steady-state conditions, this relationship governs the spatial distribution of total hydraulic head, pore-water pressure, and seepage velocity within the dam body and its foundation.

SEEP/W is widely used to simulate seepage in porous materials for both saturated and unsaturated flow regimes. Previous studies have successfully applied SEEP/W to earth dam analyses and reported reliable predictions of the phreatic surface, seepage discharge, pore-water pressure, flow velocities, and exit gradient^43–46^. The software is particularly suitable for cases where the modeled domain contains materials with strongly contrasting hydraulic conductivities.

#### Model setup

The two-dimensional cross-section of the zoned earthfill dam was modeled by defining three regions: the impervious core (*K*_*c*_), the pervious shells (*K*_*s*_) on the upstream and downstream sides, and the permeable foundation (*K*_*f*_​) extending to a depth (*D*). The dam geometry was parameterized using the dam height (*H*), crest width (*B*), upstream slope angle (*α*), downstream slope angle (*θ*), and freeboard (*F*_*b*_)​, as shown schematically in the model layout. The permeability contrast between the core and shell was represented using the ratio (*K′*=*K*_*c*_/*K*_*s*_)​. The combinations of geometric and hydraulic parameters adopted in the numerical runs are summarized in Table 1.

**Table 1:** Geometrical and physical parameters were conducted by Khursheed et al.^41^.

Parameter	Description	Unit	Values		
***B***	Dam’s crest width	m	4	5	6
***D***	Depth of the dam’s foundation	m	20	30	40
***K***_***f***_	Hydraulic conductivity of the dam’s foundation	m/day	8.64	0.0864	0.00864
***H***	Height of the earthfill dam model	m	14	16	18
***h***	Upstream head in the reservoir	m	12, 12.50, 13, 14, 14.50, 15, 16, 16.50, 17		
***K’***	Ratio of core to shell hydraulic conductivity (*K*_*c*_/*K*_*s*_)	-	10^− 2^	10^− 4^	10^− 6^
***cotα***	Upstream slope of the dam	-	2.50	2.75	3.00
***cotθ***	Downstream slope of the dam	-	2.00	2.25	2.50

**Fig. 3:**
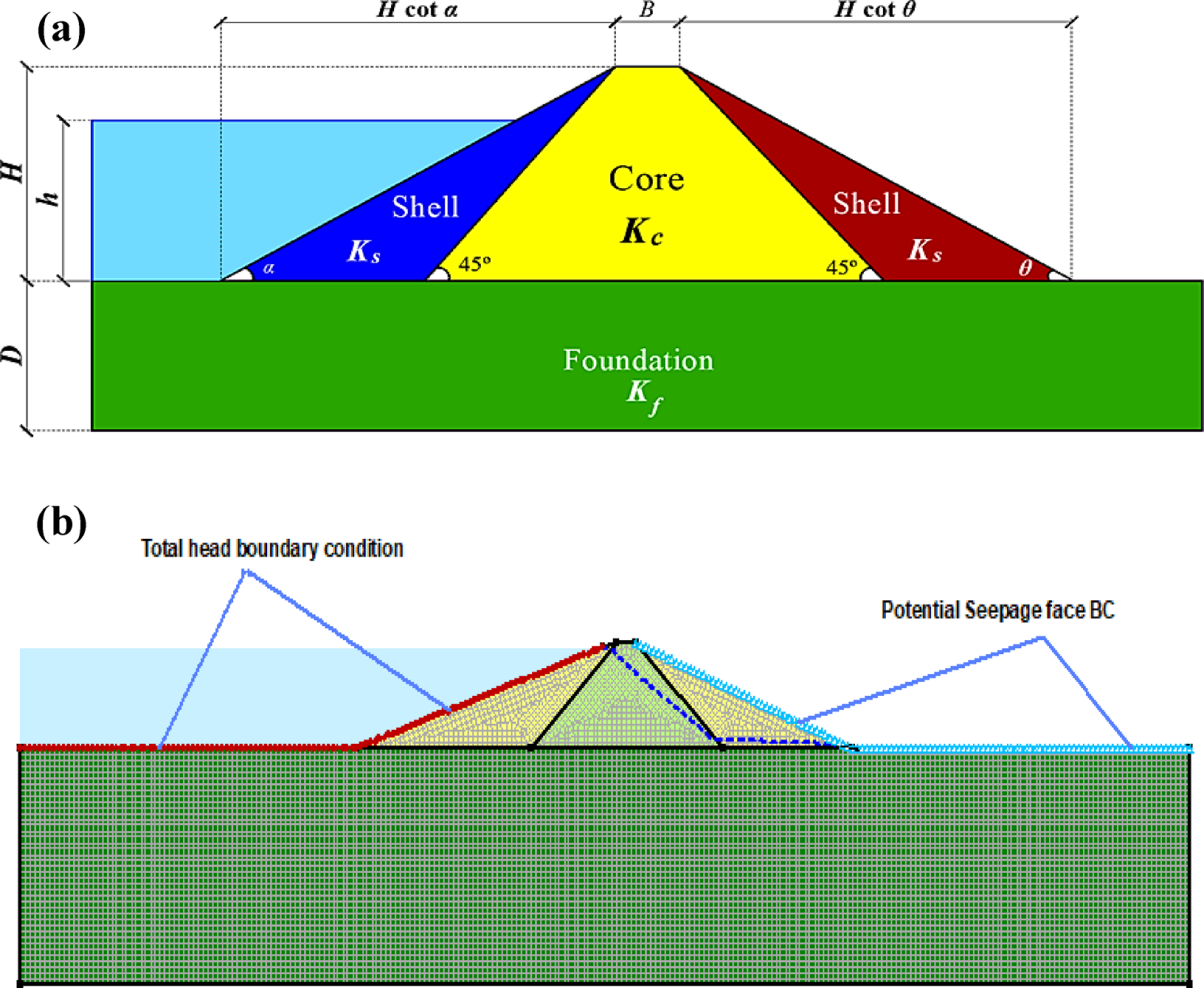
Model setup conducted by Khursheed et al.^41^: (**a**) assigned boundary conditions and locations of the flux sections; (**b**) finite-element mesh discretization.

A steady-state seepage analysis was carried out using a coupled saturated–unsaturated formulation to capture the position of the phreatic surface and the seepage discharge through the dam–foundation system. The reservoir was simulated by applying a total head boundary condition along the upstream face corresponding to the specified water level. On the downstream face, a potential seepage face boundary condition was assigned to allow the phreatic line to emerge naturally wherever pore-water pressures become zero. To quantify seepage discharge, flux sections were defined at selected locations along the downstream side (Fig. 3a). The domain was discretized using a finite-element mesh with finer elements in the dam body and near material interfaces, and comparatively coarser elements in the field foundation (Fig. 3b).

### Problem formulation

In this study, seepage discharge per unit dam length (*q*) is formulated as a function of the governing geometric and hydraulic parameters describing a heterogeneous earthfill dam founded on a permeable foundation. Based on the adopted dam configuration (Fig. 3) and the available input descriptors, the relationship can be expressed as:

2 $$\:q=\phi\:\left(B,\:D,h,Hcot\alpha\:,Hcot\theta\:,\:{K}^{{\prime\:}},{K}_{f}\right)$$

Where $\:\phi\:$ is a functional symbol; $\:Hcot\alpha\:$ is the horizontal length of upstream slope dam, $\:Hcot\theta\:$ is the horizontal length of downstream slope dam. This functional form defines the prediction problem addressed in the subsequent sections, where *q* is modeled from the above inputs using baseline regression and ML approaches.

#### Descriptive statistics

**Fig. 4:**
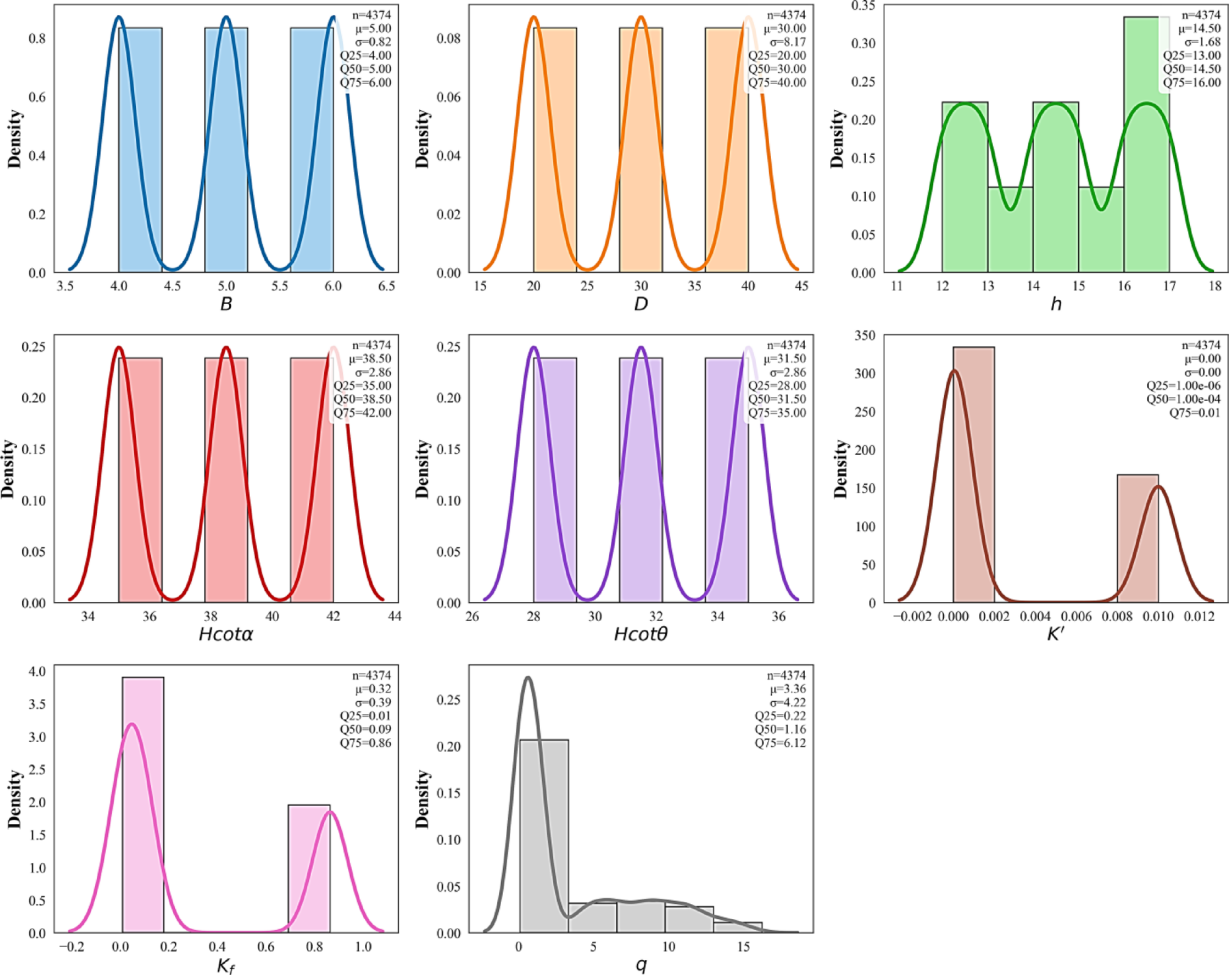
KDE plots for the investigated parameters in the collected dataset.

Figure 4 presents a set of Kernel Density Estimation (KDE) plots that show the distribution of various parameters from the collected dataset. The plots give insights into the underlying distribution of each parameter and allow for a deeper understanding of their characteristics. For each variable, the mean ($\:\mu\:$), 25th percentile (Q_25_), median (Q_50_), and 75th percentile (Q_75_) are reported to provide a concise quantitative description of central tendency and dispersion. The geometric parameters (*B*, *D*, and *h*) exhibit multi-modal distributions, reflecting the discrete combinations of parameter values adopted in numerical simulations. Similar distributional behavior is observed for the transformed slope parameters ($\:Hcot\alpha\:$ and $\:Hcot\theta\:$), which arise from the systematic variation of dam height and slope angles. The multi-modal nature of these distributions reflects the discrete combinations of dam height and slope angles adopted in parametric study.

The geometric parameters exhibit distinct yet interpretable variability patterns. $\:B$ shows a mean value of $\:\mu\:=5.0$m and an Interquartile Range (IQR = Q_75_ − Q_25_) of 2.0 m. $\:D$ displays substantially higher variability, with a mean of $\:\mu\:=30.0$m and an IQR of 20.0 m, reflecting the wide range of foundation conditions considered in the numerical simulations. $\:h$ has a mean of $\:\mu\:=14.5$m and a moderate IQR of 3.5 m, indicating controlled variation around typical reservoir operating levels. $\:Hcot\alpha\:\:$and $\:Hcot\theta\:$ exhibit comparable dispersion, with mean values of $\:\mu\:=38.5$ and $\:\mu\:=31.5$, respectively, and identical IQRs of 7.0, highlighting balanced variability in upstream and downstream slope geometries.

Hydraulic parameters show the most pronounced heterogeneity. $\:{K}^{{\prime\:}}$ exhibits a near-zero mean due to its logarithmic-scale representation and a small absolute IQR on the order of $\:{10}^{-4}$; however, this narrow numerical range corresponds to permeability contrasts spanning several orders of magnitude. $\:{K}_{f}$ demonstrates substantial dispersion, with a mean value of $\:\mu\:=0.32$ and a wide IQR of 0.85, emphasizing the strong variability in foundation permeability conditions. Finally, $\:q$ exhibits a mean value of $\:\mu\:=3.36$ m³/d/m and a large IQR of 5.90 m³/d/m, along with a pronounced right-skewed distribution. This behavior reflects the nonlinear response of seepage flux to interacting geometric and hydraulic controls and highlights the presence of extreme seepage scenarios. Overall, the wide range of mean values, interquartile spreads, and distribution shapes confirms a highly heterogeneous and non-Gaussian feature space, reinforcing the suitability of nonlinear and ensemble-based ML models for accurately capturing seepage behavior in earthfill dams.

#### Correlation analysis

To rigorously evaluate the relationships between the predictor variables and the response variable, both Pearson and Spearman correlation analyses were employed. The combined use of these metrics ensures robustness against deviations from linearity, non-normality, and the presence of outliers. The Pearson correlation coefficient (*r*) quantifies the strength and direction of linear relationships and is defined as^47^:

3 $$\:r=\frac{{\sum\:}_{i=1}^{n}({x}_{i}-\stackrel{̄}{x})({y}_{i}-\stackrel{̄}{y})}{\sqrt{{\sum\:}_{i=1}^{n}({x}_{i}-\stackrel{̄}{x}{)}^{2}{\sum\:}_{i=1}^{n}({y}_{i}-\stackrel{̄}{y}{)}^{2}}}$$

where *x*_*i*_ and *y*_*i*_ are individual observations, $\:\stackrel{̄}{x}$ and $\:\stackrel{̄}{y}$ are sample means, and *n* is the number of observations. On the other hand, the Spearman rank correlation coefficient ($\:\rho\:$) is a non-parametric measure of monotonic association, which is defined as:

4 $$\:\rho\:=1-\frac{6{\sum\:}_{i=1}^{n}{d}_{i}^{2}}{n({n}^{2}-1)}$$

where *d*_*i*_ is the difference between the ranks of paired observations. Spearman correlation is insensitive to distributional assumptions and is particularly robust in the presence of nonlinear but monotonic relationships. The agreement between Pearson and Spearman correlation coefficients was used as an indicator of the stability and reliability of the identified relationships.

### Overview of ML models

In this study, the ML models were developed using Python within the Anaconda software environment, which offers a comprehensive suite of tools and libraries, such as NumPy, pandas, and scikit-learn, that are crucial for building and deploying ML models^48^. The models were selected based on their performance, interpretability, and ability to handle various types of data. The integration of these models within Anaconda ensures a reproducible workflow, facilitating consistent experimentation and easy deployment.

The selection of the five ML models was guided by the nonlinear and heterogeneous nature of seepage processes in earthfill dams, where complex interactions exist between hydraulic loading, material properties, and geometry. The DT model was included for its transparency and ability to capture nonlinear threshold-based relationships that commonly arise in hydrogeological systems in an interpretable manner^49^. The RF model, as an ensemble of decision trees, was adopted to improve robustness by averaging multiple nonlinear models, which is well suited for handling spatial variability and parameter uncertainty in seepage-related datasets^50^.

The SGB and LGB models were selected due to their ability to sequentially refine predictions by focusing on residual errors, enabling them to better capture subtle interactions and localized effects that govern seepage flow in heterogeneous dam–foundation systems. These properties often allow gradient boosting methods to outperform the RF model in tabular regression problems involving complex nonlinear dependencies^51^. Finally, the CGB model was included for its efficient handling of feature interactions and regularization, which reduces overfitting while maintaining high predictive accuracy^52^. Collectively, these models provide a diverse yet complementary set of approaches for modeling nonlinear hydrogeological behavior, enabling a comprehensive evaluation of predictive performance and generalization.

### Model optimization

Bayesian optimization (BO) was employed for hyperparameter tuning under a limited evaluation budget, where the objective function (prediction error) is expensive to compute and potentially nonconvex and noisy. BO follows a sequential model-based optimization framework in which a probabilistic surrogate model is fitted to previously evaluated hyperparameter configurations and then used to propose new candidates through an acquisition function that balances exploration and exploitation. This approach is widely used for black-box optimization and machine-learning hyperparameter tuning. The BO procedure was configured to run for 100 iterations (N_ITER = 100). Candidate configurations were evaluated using 5-fold cross-validation (OPT_CV = 5), where the training data were partitioned into five folds, and the mean cross-validated RMSE (CV-RMSE) was computed as the optimization objective. This resampling strategy reduces variance in performance estimation compared with a single split and provides a more stable basis for model selection.

After completion of the search, model selection was not restricted to the single configuration achieving the minimum CV-RMSE. Instead, the TOP_N = 30 configurations with the smallest CV-RMSE values were retained to form a high-performing candidate set, which reduces sensitivity to small fluctuations among near-optimal candidates and aligns with robust Auto ML practices that combine BO with downstream selection procedures^53^. Final hyperparameter selection was performed using an overfitting-aware criterion that jointly considers validation accuracy and the train–validation generalization gap. For each retained configuration, Train-RMSE was computed, and a penalized score was formed as: $\:\rm{Penalized\,RMSE}=\mathrm{CV-RMSE}+\alpha\:{max}\:(0,\mathrm{CV-RMSE}-\mathrm{Train-RMSE})$, with overfit alpha (α) equals 1. The penalty activates only when Train-RMSE is substantially lower than CV-RMSE, indicating an inflated generalization gap consistent with overfitting during model selection. This regularization of the selection criterion is consistent with established findings on overfitting in model selection and selection bias in performance evaluation^54^.

**Fig. 5:**
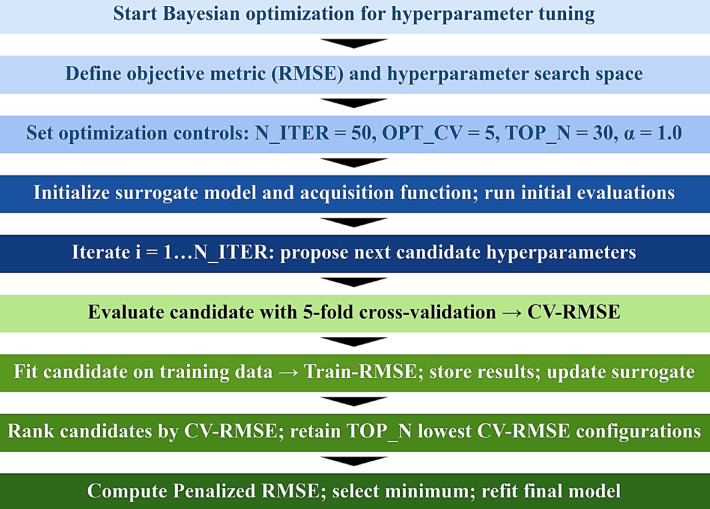
Methodology of the BO hyperparameter tuning process.

Following selection of the final hyperparameter configuration, performance estimation and prediction extraction were repeated using both 5-fold and 10-fold CV to assess robustness of the reported error and stability of cross-validated predictions under an alternative resampling scheme. The 10-fold cross-validation results were treated as a supplementary robustness check rather than the objective guiding the Bayesian optimization loop^55^. The full BO pipeline and the post-selection evaluation step are summarized in Fig. 5.

### Prediction accuracy evaluation

Evaluation of proposed models follows a comprehensive framework that combines visualization and quantitative metrics to ensure thorough performance assessment^56^. Scatter plots are used to visually compare actual and predicted values, helping to identify model accuracy and potential systematic errors, while Residual Error Curves (REC) assess performance across different error thresholds by showing the percentage of predictions within acceptable limits^57^. In addition, several regression metrics are applied to quantify model accuracy and reliability, with the corresponding equations presented in Table 2. To ensure robust evaluation, prediction reliability is also assessed through the 95% confidence interval (*U*_*95*_), which combines standard deviation (SD) and RMSE to highlight the expected variability in model outputs.

**Table 2:** Selected performance assessment metrics.

Regression metric	Equation
**Determination coefficient**	$\:{\mathrm{R}}^{2}=1-{\sum\:}_{i=1}^{n}{\left({y}_{i}-\widehat{{y}_{i}}\right)}^{2}/{\sum\:}_{i=1}^{n}{\left({y}_{i\:}-\stackrel{-}{y}\right)}^{2}$
**Root Mean Squared Error**	$$\:\mathrm{R}\mathrm{M}\mathrm{S}\mathrm{E}=\sqrt{\raisebox{1ex}{$\sum\:_{i=1}^{\:n}{\left({y}_{i}-\widehat{{y}_{i}}\right)}^{2}$}\!\left/\:\!\raisebox{-1ex}{$n$}\right.\:}$$
**Root Mean Squared Relative Error**	$\:\mathrm{R}\mathrm{M}\mathrm{S}\mathrm{R}\mathrm{E}=\sqrt{\frac{1}{n}\sum\:_{i=1}^{\:n}{\left(\frac{{y}_{i}-\widehat{{y}_{i}}}{{y}_{i}}\right)}^{2}}$
**Mean Absolute Error**	$\:\mathrm{M}\mathrm{A}\mathrm{E}=\frac{\sum\:_{i=1}^{\:n}\left|{y}_{i}-\widehat{{y}_{i}}\right|}{n}$
**Mean Absolute Relative Error**	$\:\mathrm{M}\mathrm{A}\mathrm{R}\mathrm{E}=\frac{\sum\:\left|\frac{{y}_{i}-\widehat{{y}_{i}}}{{y}_{i}}\right|}{n}$
**Percent Bias**	PBIAS = $\:\frac{\sum\:_{i=1}^{n}\left(\widehat{{y}_{i}}-{y}_{i}\right)}{n}$
**Uncertainty Measure**	*U*_*95*_ =1.96$\:\sqrt{{\mathrm{R}\mathrm{M}\mathrm{S}\mathrm{E}}^{2}+{\mathrm{S}\mathrm{D}}^{2}}$

## Results and discussion

### Correlation exploration of dataset

**Fig. 6:**
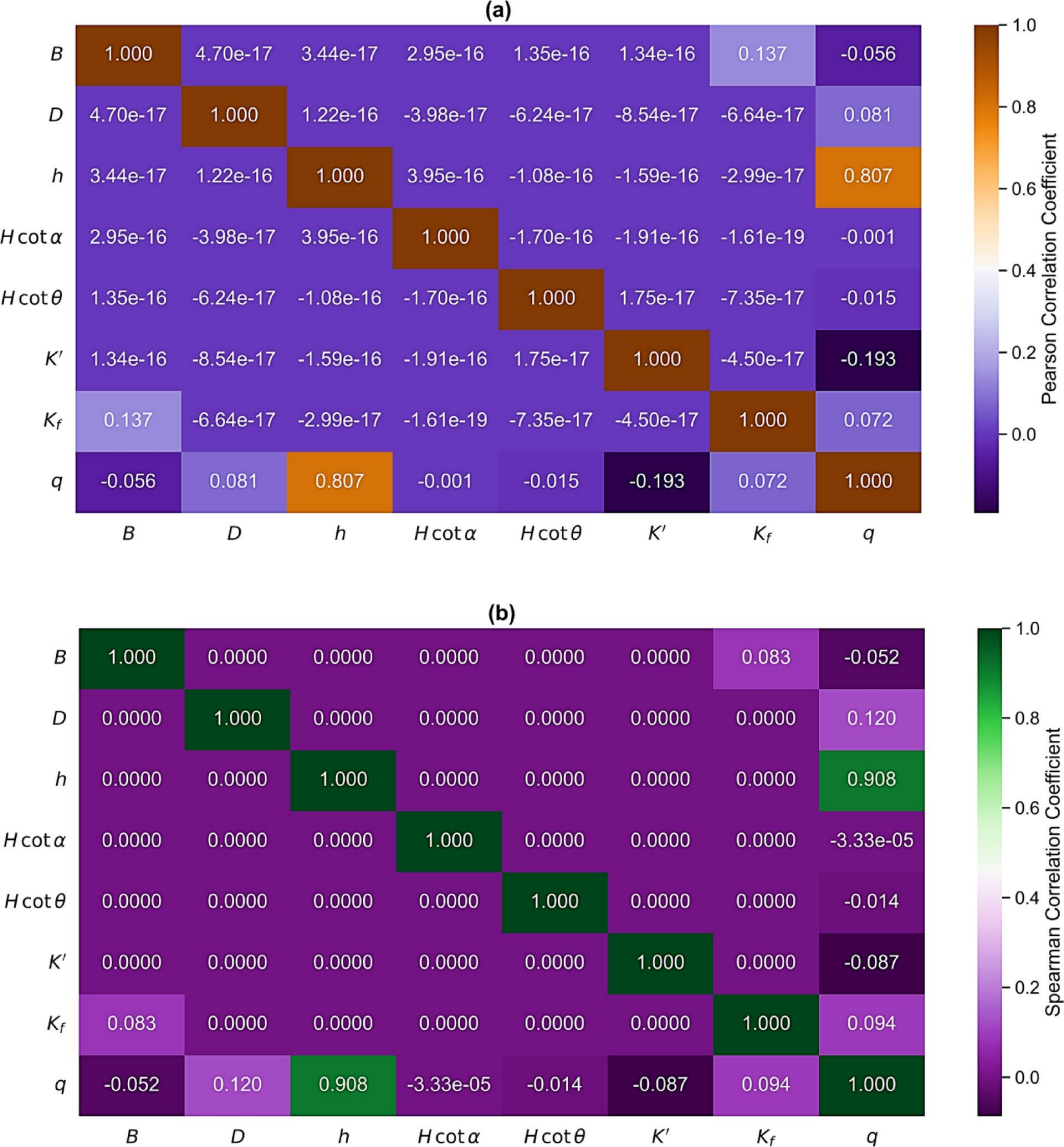
Heatmaps between studied variables based on **(a)** Pearson and **(b)** Spearman correlation coefficients.

Figure 6 illustrates the correlation structure among the studied input variables and the seepage discharge output using Pearson and Spearman correlation coefficients, respectively. The Pearson correlation heatmap (Fig. 6a) reveals that the upstream water head exhibits a strong positive linear correlation with seepage discharge, with a correlation coefficient of $\:r=0.807$. This indicates that increases in reservoir head lead to a proportional increase in seepage discharge, consistent with classical seepage theory. Meanwhile, all remaining geometric parameters (*B*, *D*, *Hcotα* and *Hcotθ*), display negligible linear correlations with seepage discharge ($\:\mid\:r\mid\:<0.10$). Similarly, $\:{K}^{{\prime\:}}$ shows a weak negative correlation ($\:r=-0.193$), while $\:{K}_{f}$ exhibits a very weak positive correlation ($\:r=0.072$). The near-zero off-diagonal values among the input variables further indicate a low degree of multicollinearity, confirming that the selected predictors are largely independent and suitable for regression-based modeling.

The Spearman rank correlation heatmap (Fig. 6b) confirms the findings of the Pearson analysis. $\:h$ again demonstrates a strong monotonic relationship with seepage discharge, with a Spearman coefficient of $\:\rho\:=0.908$, reinforcing its dominant influence on seepage behavior. All other predictors exhibit weak or negligible monotonic correlations with seepage discharge ($\:\mid\:\rho\:\mid\:<0.15$), including the slope parameters and hydraulic conductivity descriptors. As a result, variables exhibiting strong Pearson correlations also demonstrate comparable Spearman coefficients, indicating that the dominant relationships are both linear and monotonic. This agreement confirms that the observed dependencies are not driven by outliers or nonlinear artifacts, thereby supporting the statistical robustness of the dataset. Moreover, the low inter-variable correlations suggest minimal redundancy among predictors, justifying the applicability of linear regression-based models, while still allowing more advanced ML approaches to capture higher-order interactions.

### Baseline linear regression model

A multiple linear regression (MLR) model was developed using the seven governing physical and geometrical variables to establish a baseline parametric relationship for seepage discharge prediction^58^. The model achieved a coefficient of determination of $\:{R}^{2}=0.704$, indicating that 70.4% of the variability in seepage discharge is explained by the selected predictors. The equality between the adjusted and unadjusted $\:{R}^{2}$ values confirm that the model does not suffer from overfitting, despite the inclusion of multiple explanatory variables. Additionally, the interpretation of the F-statistic and *p*-value in MLR was tested. The F-statistic tests the joint significance of all predictors, while the associated *p*-value quantifies the probability that the observed regression relationship occurred by chance; the large F-value and $\:p<0.05$ confirm the strong statistical significance of the model. In the present study, the MLR model is highly statistically significant ($\:F=1484$, $\:p<0.001$), demonstrating that the observed relationship between the predictors and the response variable is extremely unlikely to have occurred by random chance. The resulting regression equation (Eq. 5) is expressed as follows:

5 $$\:q=-24.4-0.348\:B+0.0416\:D+2.02\:h-0.0016\:\left(Hcot\:\alpha\:\right)-0.0216\:\left(Hcot\:\theta\:\right)-173.1\:{K}^{{\prime\:}}+0.892\:{K}_{f}$$

Table 3 summarizes the standard errors, *t*-statistics, *p*-values, 95% confidence intervals (CI), and variance inflation factors (VIFs) for all predictors. The upstream water head emerges as the most influential predictor, exhibiting a large positive coefficient (2.020) with a very high *t*-value (97.99, $\:p<0.001$). This finding is fully consistent with seepage theory and the correlation analysis presented earlier. $\:D$ and $\:{K}_{f}$ also show statistically significant positive effects ($\:p<0.001$), indicating that deeper and more permeable foundations contribute to increased seepage rates. Conversely, $\:{K}^{{\prime\:}}$ exhibits a strong negative coefficient (− 173.09, $\:p<0.001$), reflecting the effectiveness of a low-permeability core in reducing seepage discharge.

Among the geometric descriptors, the crest width has a statistically significant negative influence on seepage, suggesting that wider crests increase seepage flow paths and hydraulic resistance. In contrast, $\:Hcot\:\alpha\:$ and $\:Hcot\:\theta\:$ are statistically insignificant ($\:p>0.05$), indicating a limited linear contribution to seepage discharge within the investigated parameter ranges. All predictors exhibit VIF values close to unity, confirming the absence of multicollinearity and supporting the numerical stability and interpretability of the regression model.

**Table 3:** Statistical significance of the MLR model.

Variable	Std. Error	t-value	*p*-value	95% CI	VIF
*q*	0.719	-33.93	< 0.001	[-25.82, -22.99]	-
*B*	0.043	-8.1	< 0.001	[-0.432, -0.264]	1.02
*D*	0.004	9.79	< 0.001	[0.033, 0.050]	1
*h*	0.021	97.99	< 0.001	[1.980, 2.061]	1
*Hcotα*	0.012	-0.13	0.896	[-0.025, 0.022]	1
*Hcotθ*	0.012	-1.78	0.076	[-0.045, 0.002]	1
*K’*	7.399	-23.39	< 0.001	[-187.60, -158.58]	1
*K*_*f*_	0.091	9.83	< 0.001	[0.714, 1.070]	1.02

### Bayesian optimization

#### Train RMSE

**Fig. 7:**
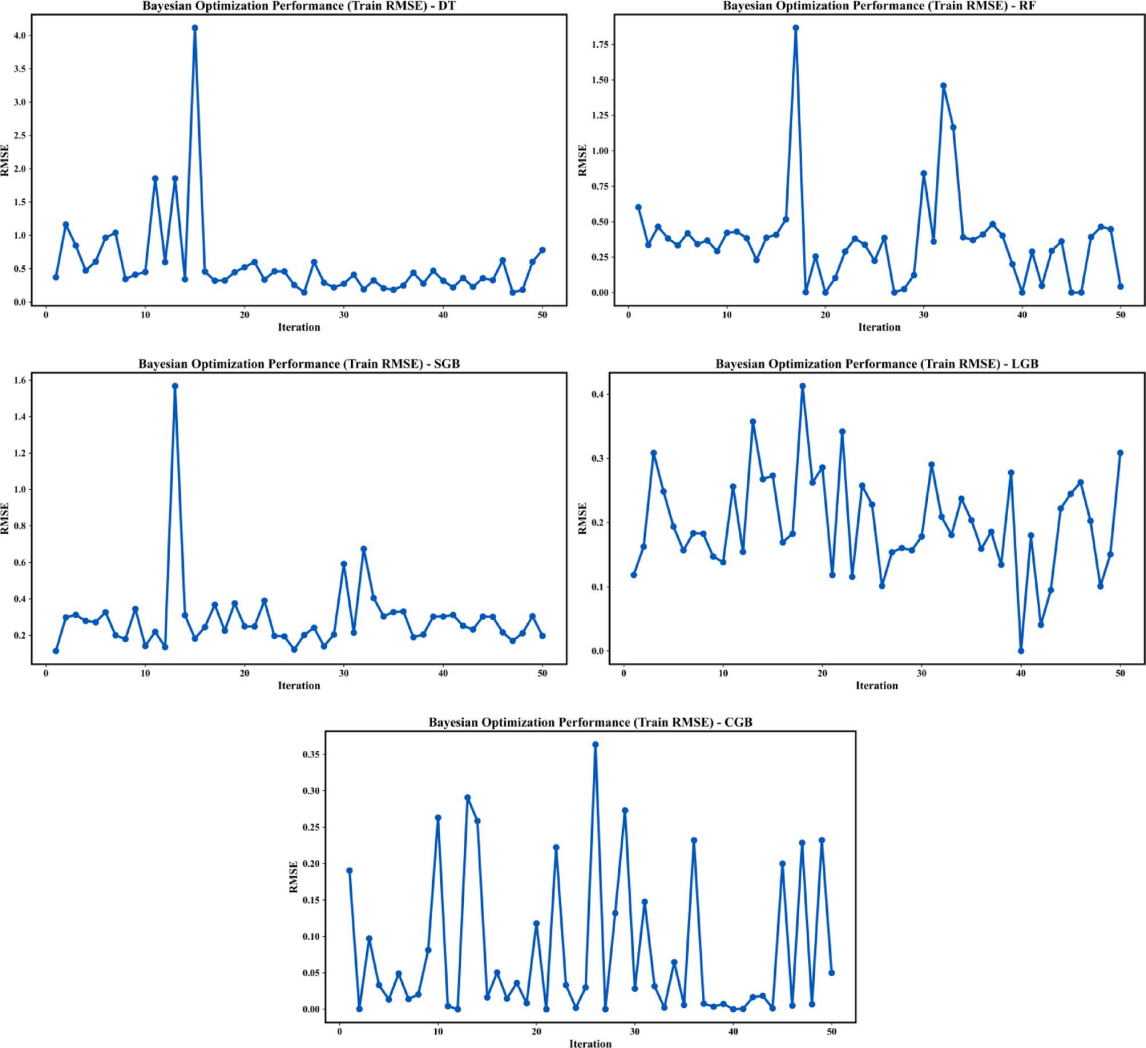
Evolution of training RMSE for BO Performance.

Figure 7 illustrates the evolution of the training RMSE over 50 BO iterations for the five investigated ML models, reflecting their ability to efficiently explore the hyperparameter space and converge toward optimal configurations. The DT model exhibits pronounced instability, with large RMSE spikes during early iterations, indicating strong sensitivity to hyperparameter selection and a tendency toward overfitting due to the absence of ensemble averaging. The RF model shows improved stability relative to the DT model, with generally lower RMSE values and reduced fluctuations, although occasional sharp increases persist, reflecting sensitivity to tree depth and ensemble size. The SGB model demonstrates a smoother convergence pattern than the DT and RF models, maintaining relatively consistent RMSE levels across most iterations; however, intermittent RMSE peaks occur during exploration phases, indicating sensitivity to learning rate and boosting depth parameters.

In contrast, the LGB model exhibits the most stable and consistently low training RMSE throughout the optimization process, with a smooth convergence trajectory and minimal extreme fluctuations. The CGB model achieves very low minimum RMSE values, with several iterations approaching near-zero error; nevertheless, its higher oscillations compared to the LGB model suggest greater sensitivity to hyperparameter choices and a potential risk of overfitting if not properly controlled. Overall, the comparative assessment indicates that boosting-based ensemble models outperform tree-based approaches, with the CGB model providing the most balanced trade-off between accuracy and stability.

#### CV RMSE

**Fig. 8:**
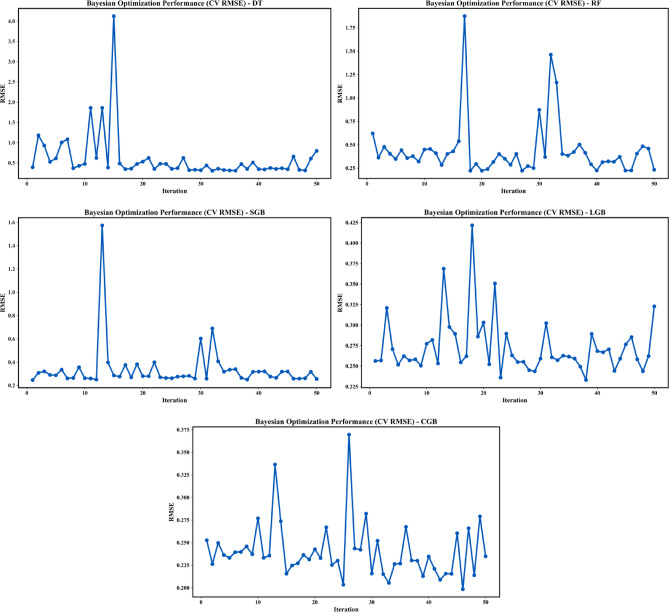
Evolution of CV RMSE for BO Performance.

Figure 8 presents the evolution of the cross-validated RMSE over 50 BO iterations for the five investigated models, providing insight into their generalization performance under varying hyperparameter configurations. The DT model again exhibits substantial variability, with pronounced RMSE spikes during early iterations, indicating strong sensitivity to hyperparameter selection and limited robustness when evaluated on unseen folds. Although CV RMSE gradually stabilizes in later iterations, the high fluctuation magnitude suggests poor generalization consistency. The RF model shows improved stability relative to the DT model, with lower average CV RMSE values; however, intermittent sharp increases remain, reflecting sensitivity to ensemble configuration and residual variance across folds.

The SGB model demonstrates a comparatively smoother CV RMSE trajectory, maintaining low error levels across most iterations, although isolated peaks appear during exploratory steps. In contrast, the LGB model exhibits the most stable CV RMSE behavior, with minimal fluctuations and consistently low error values, indicating strong generalization capability and effective regularization during hyperparameter tuning. The CGB model achieves CV RMSE values comparable to the LGB model and occasionally lower minima; however, its higher oscillations across iterations suggest greater sensitivity to hyperparameter choices. Overall, the CV RMSE analysis confirms that boosting-based ensemble models outperform tree-based approaches, with the LGB and CGB models providing most robust balance between predictive accuracy, thus minimizing the risk of overfitting.

#### Overfitting gap

**Fig. 9:**
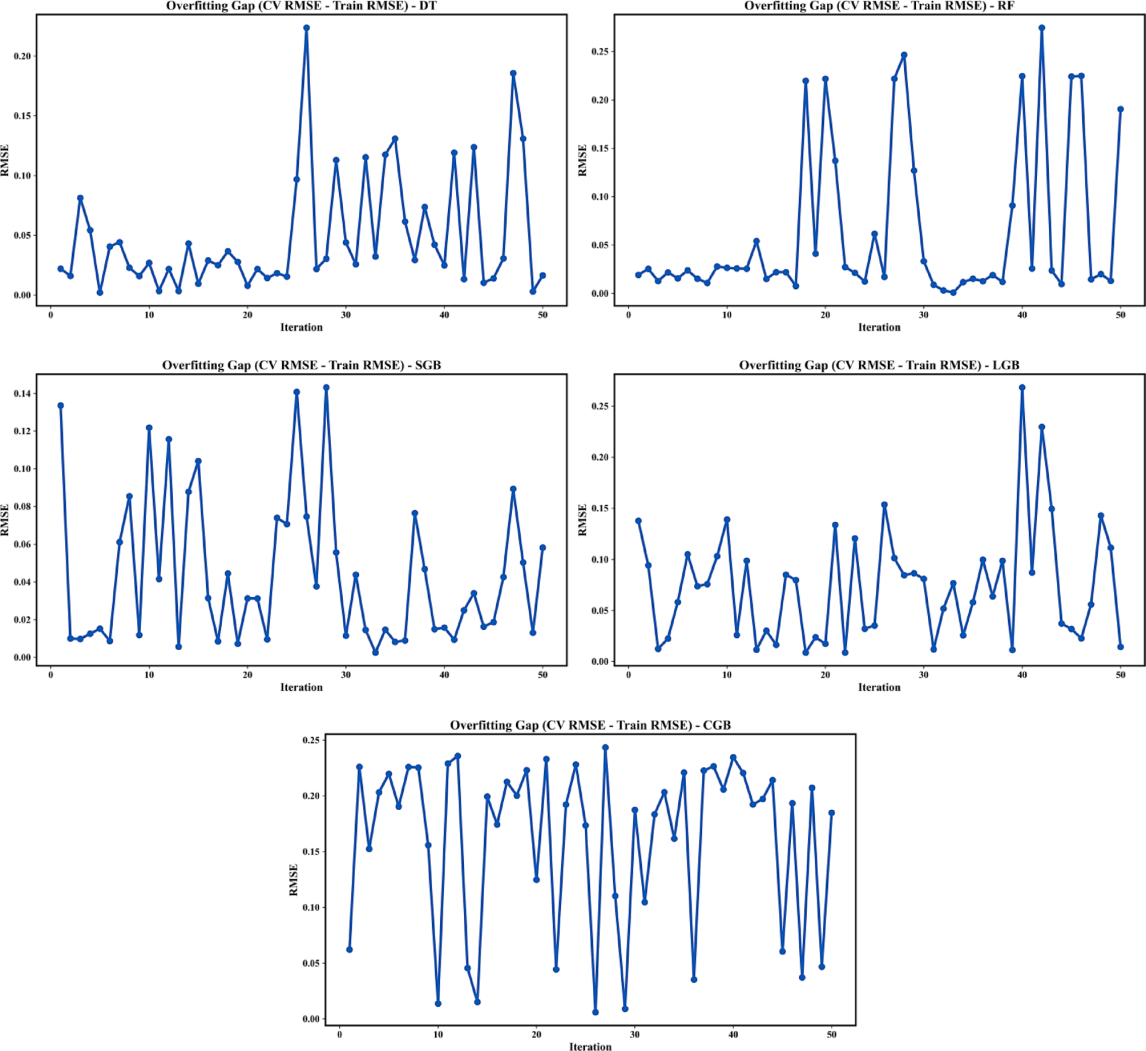
Evolution of the overfitting gap for BO Performance.

Figure 9 presents the evolution of the overfitting gap, defined as the difference between CV RMSE and training RMSE (CV RMSE − Train RMSE), across 50 BO iterations for the five investigated models. This metric provides a direct measure of each model’s tendency to overfit during hyperparameter tuning. The DT model exhibits pronounced fluctuations and several large positive gaps, particularly during early and mid-optimization stages, indicating limited generalization and strong sensitivity to hyperparameter configurations. Similarly, the RF model shows intermittent large overfitting gaps, reflecting instability in generalization performance despite ensemble averaging.

The SGB model demonstrates a comparatively smaller and more controlled overfitting gap, with most iterations remaining within a narrow RMSE range, suggesting improved generalization behavior. The LGB model exhibits the most consistently low overfitting gap, with minimal dispersion across iterations, indicating an effective balance between model complexity and regularization. In contrast, the CGB model, while achieving low training errors, shows persistently larger overfitting gaps, implying stronger sensitivity to hyperparameter choices and an increased risk of overfitting.

### Hyperparameters tuning

**Fig. 10:**
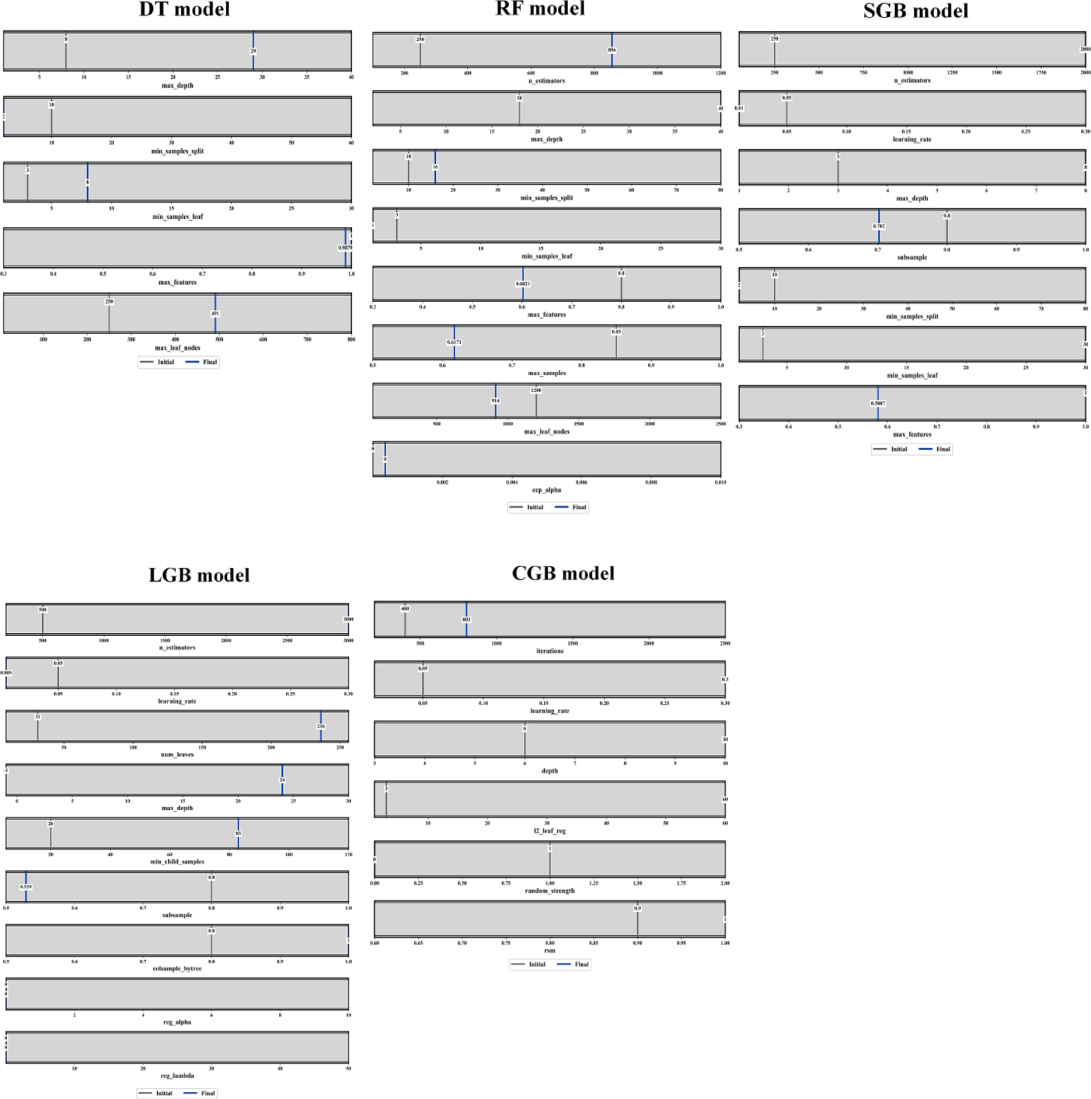
Range, initial, and final hyperparameters for developed ML models.

Figure 10 summarizes the hyperparameter tuning outcomes for all investigated models, showing the search ranges, initial reference values, and final optimized hyperparameters selected through BO. For the DT model, the optimization favored a substantially deeper tree structure, with the maximum depth increasing from an initial value of 8 to a final value of 29, alongside an increase in maximum leaf nodes from 250 to 491. At the same time, higher values of minimum samples per leaf (from 3 to 8) and minimum samples per split (from 10 to 2) were selected, indicating a trade-off between model expressiveness and regularization. The final max_features value converged close to unity (≈ 0.99), allowing the tree to exploit nearly all predictors at each split. For the RF model, BO substantially increased the ensemble size, selecting 856 trees compared to an initial value of 250, while also increasing the maximum tree depth from 18 to 40. Regularization was enforced through moderate increases in min_samples_split (10→16) and min_samples_leaf (3→1), and by reducing max_features from 0.8 to approximately 0.60 and max_samples to around 0.62, thereby enhancing diversity among trees. The cost-complexity pruning parameter (ccp_alpha) converged toward zero, indicating that explicit pruning was less critical once ensemble averaging was established.

The SGB model converged toward a configuration characterized by a large number of estimators (≈ 2000) combined with a low learning rate (≈ 0.01), reflecting the classical boosting trade-off between incremental learning and model stability. The optimized tree depth increased from 3 to 8, while subsampling was reduced from 0.8 to approximately 0.70, introducing stochasticity to mitigate overfitting. Moderate values of min_samples_split (10) and min_samples_leaf (3) were retained, and max_features converged to approximately 0.58, indicating partial feature utilization at each split. For the LGB model, BO selected a high-capacity yet strongly regularized configuration. The number of estimators increased markedly from 500 to 3000, while the learning rate decreased from 0.05 to 0.005, ensuring stable convergence. Model complexity was controlled through a substantial increase in num_leaves (31 → 236) and max_depth (− 1 → 24), accompanied by a strong increase in min_child_samples (20 → 83). Both subsample and colsample_bytree converged to values around 0.53–0.80, promoting randomness and robustness. Regularization terms (reg_alpha and reg_lambda) remained near zero, suggesting that structural constraints dominated over explicit penalization. Finally, the CGB model converged toward 803 boosting iterations, a relatively high learning rate (≈ 0.30), and a tree depth of 6.0, indicating a preference for moderately deep trees with faster learning. Strong regularization was achieved through an increase in L2_leaf_regularization (3 → 60) and stabilization via random strength = 1 and rsm ≈ 0.9, ensuring robustness against noise and feature dominance.

### Cross-validation analysis

**Fig. 11:**
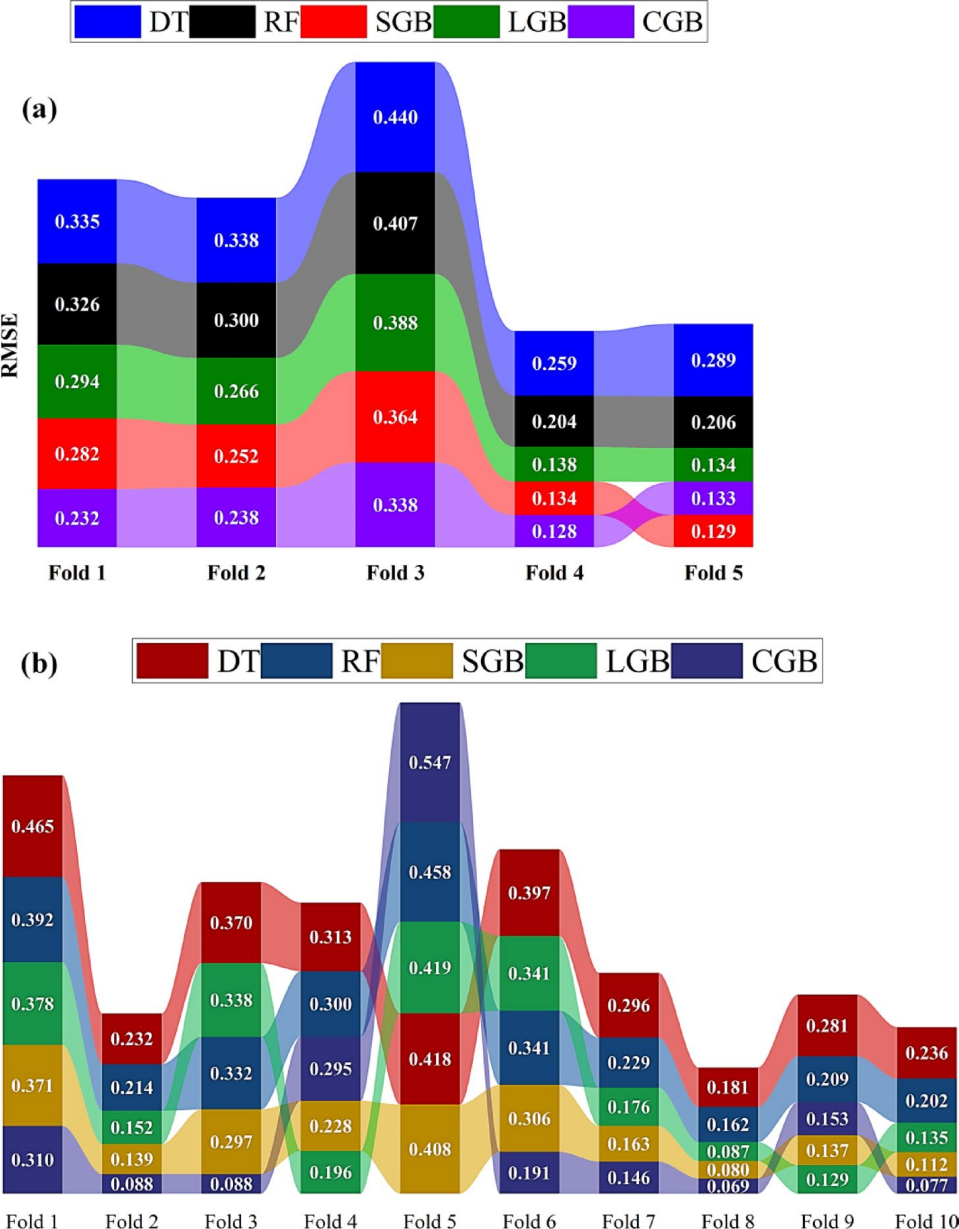
Performance of the ML models across **(a)** 5-fold and **(b)** 10-fold CV folds based on RMSE.

Figure 11 compares RMSE values for the adopted models across five-fold and ten-fold schemes. Under the five-fold CV (Fig. 11a), the SGB and CGB models consistently achieve the lowest RMSE values across all folds, demonstrating superior predictive accuracy relative to the other approaches. Among these, the CGB model generally outperforms the SGB model, attaining the minimum RMSE in most folds and exhibiting strong fitting capability. The LGB model follows closely, maintaining competitive RMSE values with slightly higher dispersion than SGB and CGB. In contrast, the RF model shows moderate performance, while the DT model records the highest RMSE values and the largest inter-fold variability, indicating limited generalization ability. This behavior is further supported by the fold-wise RMSE ranges, which span 0.129–0.364 m^3^/d/m for SGB and 0.128–0.338 m^3^/d/m for CGB, compared to substantially wider ranges for the RF model (0.204–0.407 m^3^/d/m) and DT model (0.259–0.440 m^3^/d/m), confirming the superior accuracy and stability of boosting-based models under five-fold validation.

Under a ten-fold CV (Fig. 11b), a fold-dependent behavior similar to that observed in the five-fold case is identified, with clearer distinctions in model stability. The CGB model generally exhibits the most consistent performance across the majority of folds, achieving low RMSE values throughout the validation process; however, a pronounced degradation is observed in Fold 5, where the RMSE increases sharply to 0.547 m^3^/d/m, indicating sensitivity to that specific data partition. The SGB model follows, maintaining relatively stable and low RMSE values across folds within the range 0.080–0.408 m^3^/d/m, demonstrating strong overall generalization with fewer extreme deviations. The LGB model ranks third, showing competitive and consistent performance (0.087–0.419 m^3^/d/m) that is broadly comparable to the RF model (0.162–0.458 m^3^/d/m), both exhibiting moderate fold-to-fold variability. Finally, the DT model consistently records the highest RMSE values (0.181–0.465 m^3^/d/m) and the largest sensitivity to fold selection, confirming its comparatively weak generalization capability.

### Assessment of ML models

#### Goodness of fit

**Fig. 12:**
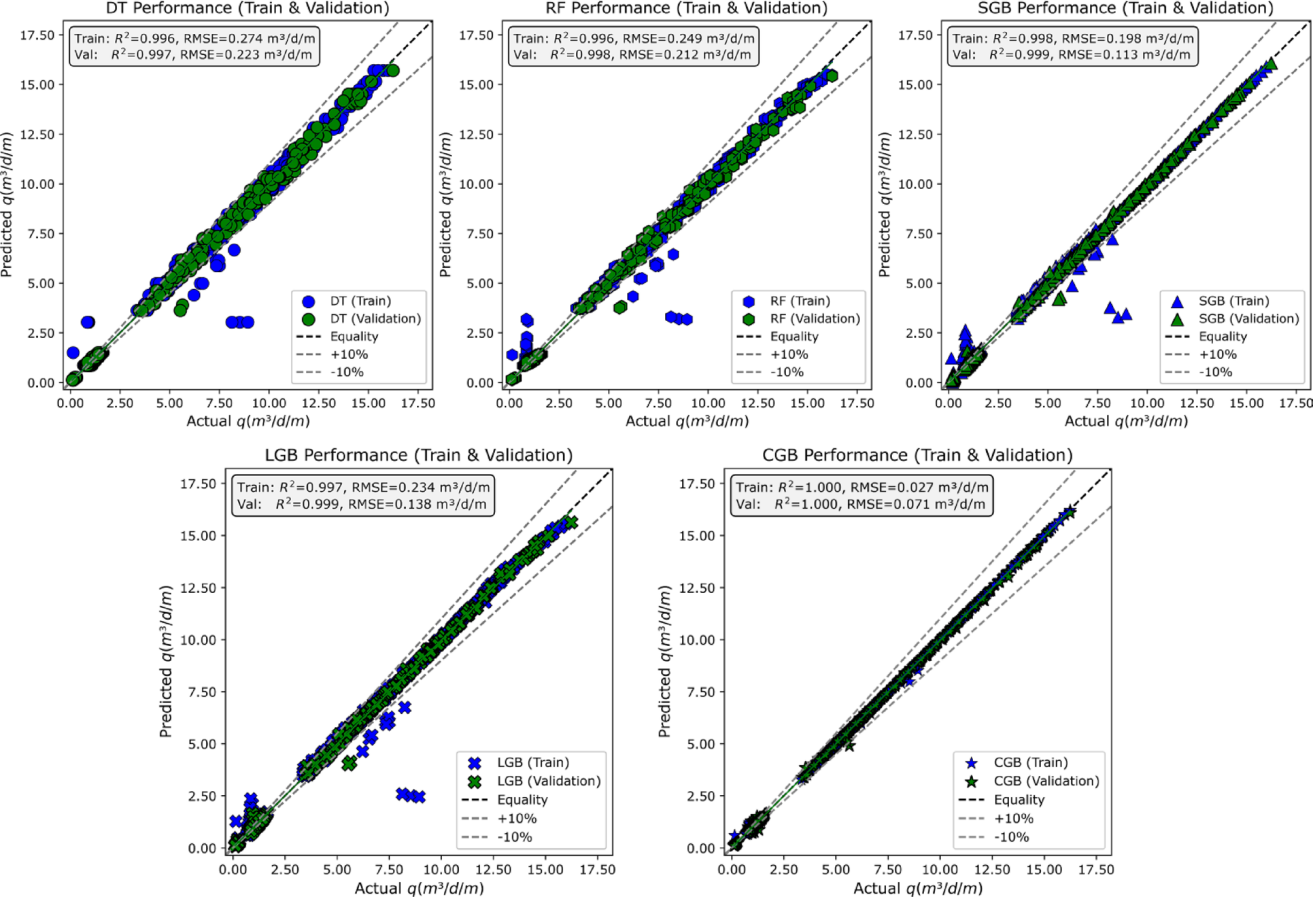
Scatter plots of predicted versus actual seepage discharge during the training and validation stages.

Figure 12 presents scatter plots of the predicted versus actual seepage discharge (*q*) for the adopted models. Each subplot shows both the training (blue markers) and validation (green markers) data. The dashed black line represents the line of equality (perfect prediction), while the grey dashed lines indicate ± 10% deviation, giving a visual reference for prediction accuracy. For the DT model, approximately 80–85% of the training data points and 85–90% of the validation points lie within the ± 10% limits. The remaining points, particularly at low and intermediate seepage discharges, exhibit noticeable deviations, which is consistent with the comparatively higher RMSE values obtained for DT (training RMSE ≈ 0.274 m³/d/m; validation RMSE ≈ 0.223 m³/d/m). The RF model shows the same behavior as the RF, as several deviations remain visible at lower and intermediate discharge levels. This behavior aligns RMSE values (training RMSE ≈ 0.25 m³/d/m; validation RMSE ≈ 0.212 m³/d/m), reflecting improved performance than the DT model but still moderate goodness of fit.

In contrast, the SGB model exhibits a marked enhancement in predictive accuracy, with approximately 92–95% of both training and validation data points falling within the ± 10% bounds. The tight clustering around the line of equality across the entire discharge range is supported by lower RMSE values (training RMSE ≈ 0.20 m³/d/m; validation RMSE ≈ 0.11 m³/d/m), indicating strong agreement between predicted and observed seepage discharges and minimal performance degradation from training to validation. The LGB model demonstrates lower behavior than the SGB model, with about 91–93% of training and validation points lying within the ± 10% limits. The dispersion is largely restricted to intermediate discharge values. This level of consistency is reflected by RMSE values for both stages (training RMSE ≈ 0.23 m³/d/m; validation RMSE ≈ 0.14 m³/d/m). The CGB model achieves the highest concentration of predictions within the ± 10% bounds, with more than 98% of training and validation points closely aligned with the equality line. The near-perfect overlap between predicted and observed values is corroborated by the exceptionally low RMSE values (training RMSE ≈ 0.03 m³/d/m; validation RMSE ≈ 0.07 m³/d/m). The small increase in RMSE from training to validation suggests slight regularization effects but no meaningful loss of accuracy, indicating excellent goodness of fit.

#### Error analysis

**Fig. 13:**
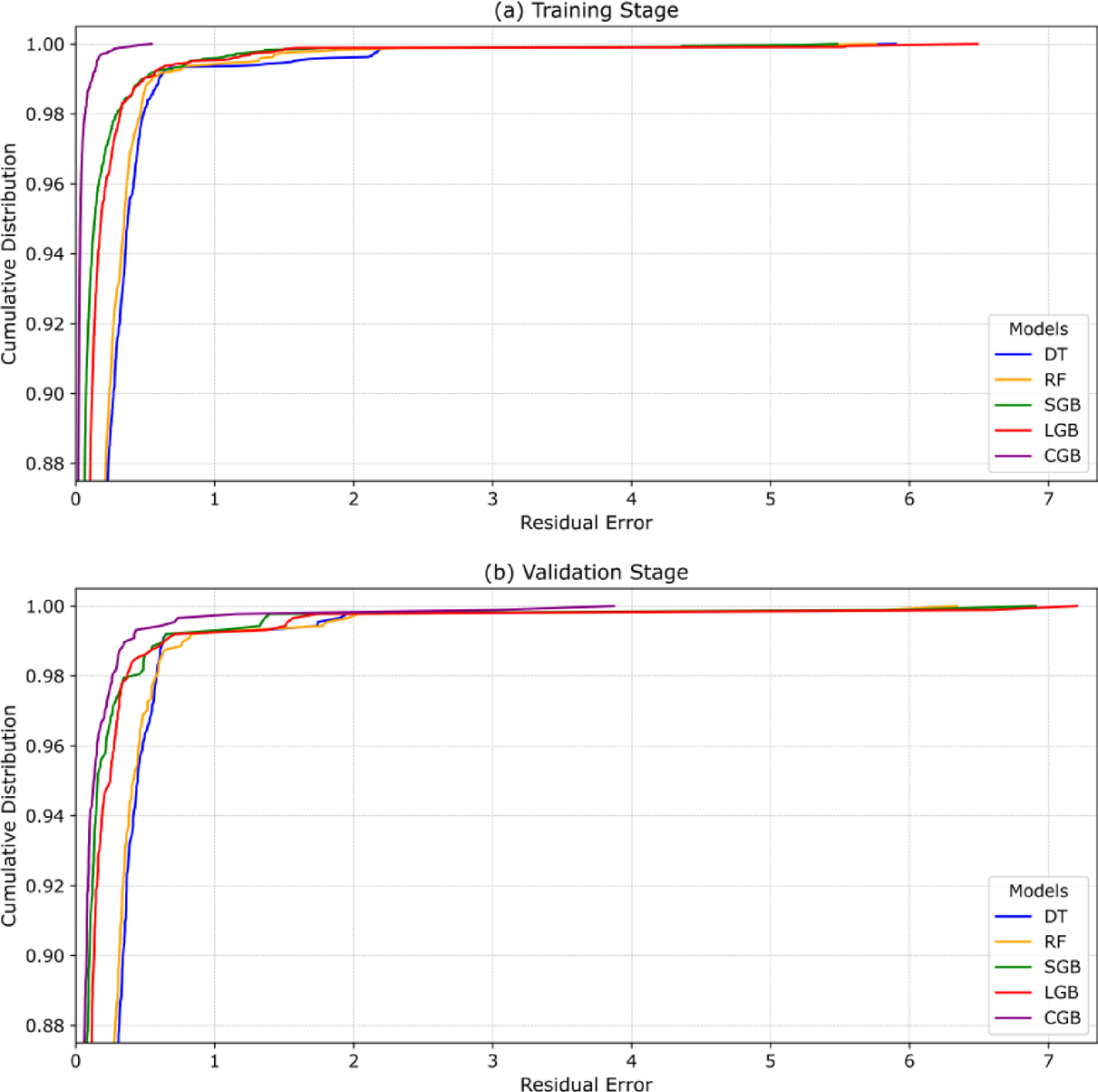
Performance of the ML models using RECs during (**a**) training and (**b**) validation stages.

Figure 13 enables a direct comparison of the predictive behavior of the investigated ML models by examining how rapidly their residual errors accumulate during the training and validation stages. In both stages, models whose REC curves rise more steeply and remain above others at low residual thresholds indicate a larger proportion of accurate predictions within tight error bounds. In the training stage (Fig. 13a), the CGB model exhibits the steepest initial rise in the low-error region, indicating that a larger fraction of their predictions falls within small residual thresholds compared to the other models. The LGB and SGB models follow closely, showing a rapid but slightly less pronounced ascent. The RF model displays a more gradual increase in cumulative probability at small residual values, while the DT model shows the slowest rise, indicating a wider spread of residual errors during training. At larger residual thresholds, except for the CGB model, the ML models converge toward similar cumulative levels, suggesting that extreme training errors are limited across approaches.

In the validation stage (Fig. 13b), the relative behavior of the models remains broadly consistent, though differences become more informative of generalization. The CGB curve continues to rise rapidly at small residual errors, maintaining a high cumulative proportion of low-error predictions, followed closely by LGB and SGB, which show comparable but slightly slower accumulation. The RF and DT models again demonstrate moderate-to-low behavior, with a noticeable delay in reaching high cumulative coverage compared to the boosting-based models. As in the training stage, all curves eventually converge at higher residual values, confirming that very large prediction errors are infrequent for all models. Overall, the REC comparison indicates that boosting-based models (CGB, LGB, and SGB) consistently achieve a higher proportion of low-residual predictions in both training and validation stages.

#### Rank analysis

Table 4 summarizes the rank-aggregation analysis of the five adopted ML models using seven statistical indices across training and validation stages. Each metric was converted to an ordinal score (1 = best & 5 = worst), and the scores were summed to provide the overall rank. The results reveal clear differences in the predictive ability and robustness of the models.

The CGB model achieves the lowest aggregated score (19) and is therefore ranked first, reflecting consistently superior performance across nearly all accuracy and reliability metrics in both training and validation stages. Notably, CGB exhibits the smallest prediction uncertainty, as indicated by its very low $\:{U}_{95}$values (0.076 in training and 0.514 in validation), confirming tight prediction intervals alongside minimal error measures. The SGB model ranks second (total score = 34) with relatively narrow uncertainty bounds ($\:{U}_{95}=0.549$ in training and 0.926 in validation). The LGB model follows in third place (total score = 45), offering competitive accuracy and stable generalization, though with moderately wider uncertainty ranges ($\:{U}_{95}=0.647$ in training and 1.009 in validation). The RF model ranks fourth (total score = 52), showing moderate predictive skill but increased uncertainty in validation ($\:{U}_{95}=1.005$), while the DT model performs weakest overall (total score = 60), characterized by the largest uncertainty bounds ($\:{U}_{95}=0.758$ in training and 1.015 in validation) and higher error levels. Collectively, the rank-aggregation results confirm that boosting-based ensemble models not only improve accuracy but also substantially reduce predictive uncertainty, with CGB providing the most reliable balance between precision and confidence in seepage discharge estimation.

Overall, the rank analysis consolidates evidence from multiple perspectives: pre- and post-tuning comparisons, cross-validation with 5 and 10 folds, scatter evaluations, and error distribution analysis. The findings converge to a clear conclusion: boosting algorithms outperform traditional tree-based models, with the CGB model offering the most accurate and stable predictions, closely followed by the SGB and LGB models, while the DT and RF models remain less reliable due to weaker generalization.

**Table 4:** Rank analysis of the adopted models.

Model	Stage	*R* ^2^	RMSE	RMSRE	MAE	MARE	PBIAS	*U*₉₅	Total Score		Rank
**DT**	Training	0.9957	0.2736	0.2245	0.1067	0.0659	3.5 × 10^− 15^	0.758	30	60	5th
	Score	(5)	(5)	(5)	(5)	(4)	(1)	(5)			
	Validation	0.9927	0.3665	0.3071	0.1313	0.0689	-0.540	1.015	30		
	Score	(5)	(5)	(3)	(5)	(3)	(4)	(5)			
**RF**	Training	0.9964	0.2494	0.2091	0.1019	0.0805	-3.0 × 10^− 14^	0.691	27	52	4th
	Score	(4)	(4)	(4)	(4)	(5)	(2)	(4)			
	Validation	0.9929	0.3629	0.2984	0.1279	0.0843	-0.5473	1.005	25		
	Score	(3)	(3)	(2)	(4)	(5)	(5)	(3)			
**SGB**	Training	0.9977	0.1983	0.1895	0.0475	0.0585	0.00627	0.549	18	34	2nd
	Score	(2)	(2)	(3)	(2)	(2)	(5)	(2)			
	Validation	0.9940	0.3343	0.3175	0.0645	0.0642	-0.4049	0.926	16		
	Score	(2)	(2)	(4)	(2)	(2)	(2)	(2)			
**LGB**	Training	0.9968	0.2336	0.1854	0.0623	0.0623	4.55 × 10^− 8^	0.647	20	45	3rd
	Score	(3)	(3)	(2)	(3)	(3)	(3)	(3)			
	Validation	0.9928	0.3641	0.3225	0.0816	0.0731	-0.4066	1.009	25		
	Score	(4)	(4)	(5)	(3)	(4)	(1)	(4)			
**CGB**	Training	0.9999	0.0274	0.0664	0.0114	0.0179	-0.0007	0.076	10	**19**	**1st**
	Score	(1)	(1)	(1)	(1)	(1)	(4)	(1)			
	Validation	0.9981	0.1860	0.2629	0.0415	0.0416	-0.4153	0.514	9		
	Score	(1)	(1)	(1)	(1)	(1)	(3)	(1)			

### SHAP analysis

**Fig. 14:**
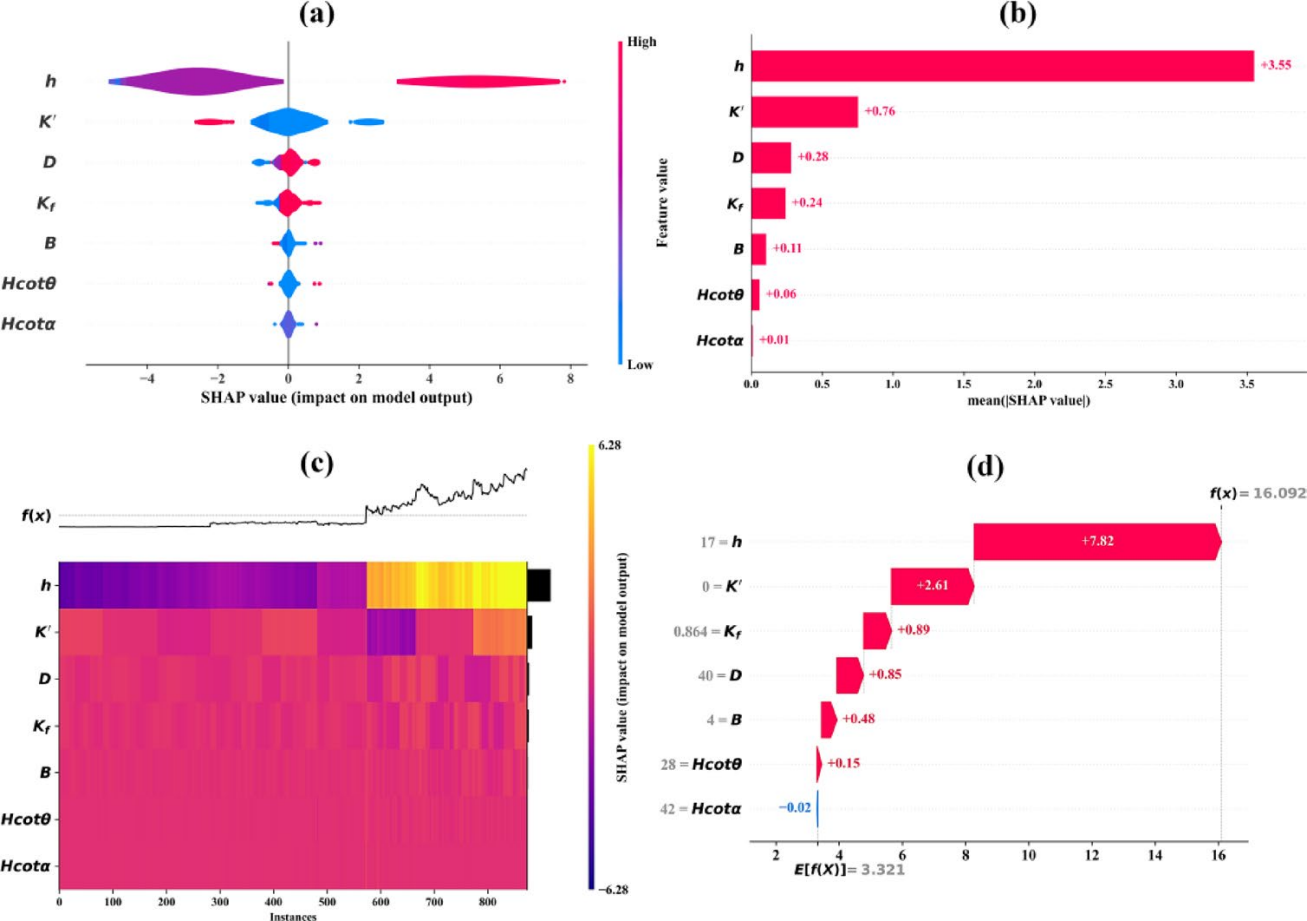
SHAP visualizations: **(a)** Summary dot plot, **(b)** Summary bar plot, **(c)** Heatmap showing the fluctuation, and **(d)** Decision plot of features contribution to the model’s predictions.

SHAP is a method that clarifies how each feature influences a model’s predictions. It calculates feature contributions, with summary plots showing overall importance and dependence plots revealing feature interactions^59^. This simplifies model interpretation, ensuring transparency and trust. Figure 14 provides a comprehensive insight into the relative importance and contribution of each input parameter to the prediction of seepage discharge across the best predictive model (CGB model).

SHAP summary dot plot (Fig. 14a) illustrates both the magnitude and direction of each input parameter’s contribution to seepage discharge predictions. Each point represents an individual scenario, colored according to the feature value (low to high). The plot shows that the upstream waterhead (*h*) has the largest influence on seepage discharge, indicating increased seepage due to elevated hydraulic gradients across the dam body and foundation. The hydraulic conductivity ratio (*K′*) also exerts a strong influence, where higher contrasts between core and shell permeability significantly alter seepage paths. The foundation permeability (*K*_*f*_) contributes positively to seepage, reflecting enhanced under-seepage through permeable foundations. Geometric parameters such as dam height (*D*) and crest width (*B*) show moderate but noticeable effects, while the slope-related parameters (*Hcotθ* and *Hcotα*) exhibit smaller and more localized contributions, indicating secondary control on seepage behavior. The SHAP summary bar plot in Fig. 14b quantifies the average contribution of each input parameter to seepage discharge prediction through their mean absolute SHAP values. The (*h*) clearly dominates the model response, with a mean SHAP value of approximately 3.55. The (*K′*) ranks second, with a mean SHAP value of about 0.76. In comparison, the remaining parameters exhibit substantially smaller contributions: (*D*) or (*K*_*f*_) show moderate influence, with mean SHAP values of approximately 0.28 and 0.24, respectively. The crest width (*B*) contributes marginally (~ 0.11), while the slope-related parameters *Hcotθ* and *Hcotα* exhibit the lowest mean SHAP values (~ 0.06 and ~ 0.01, respectively), indicating a relatively weak direct impact on seepage discharge.

On the other hand, Fig. 14c illustrates the instance-wise variation of SHAP values across the dataset, highlighting how feature contributions fluctuate from one scenario to another. The heatmap shows pronounced variability in the contribution of (*h*) and (*K′*), particularly at higher predicted seepage values, indicating strong interaction effects between hydraulic loading and material heterogeneity. In contrast, geometric parameters display more uniform and lower-magnitude contributions across instances, confirming their relatively stable but secondary role in controlling seepage discharge. Finally, Fig. 14d depicts a representative decision plot, showing how individual feature contributions accumulate from the baseline prediction to the final seepage discharge estimate. The plot demonstrates that increases in (*h*) and (*K′*) drive the prediction upward most significantly, while (*K*_*f*_ ) and (*D*) further adjust the estimate depending on foundation permeability and dam geometry. Parameters related to slopes (*Hcotθ and Hcotα*) contribute marginally, occasionally offsetting or slightly reinforcing the dominant effects. This sequential contribution illustrates how seepage discharge emerges from the combined influence of hydraulic loading, material properties, and geometry.

### Interactive GUI

**Fig. 15:**
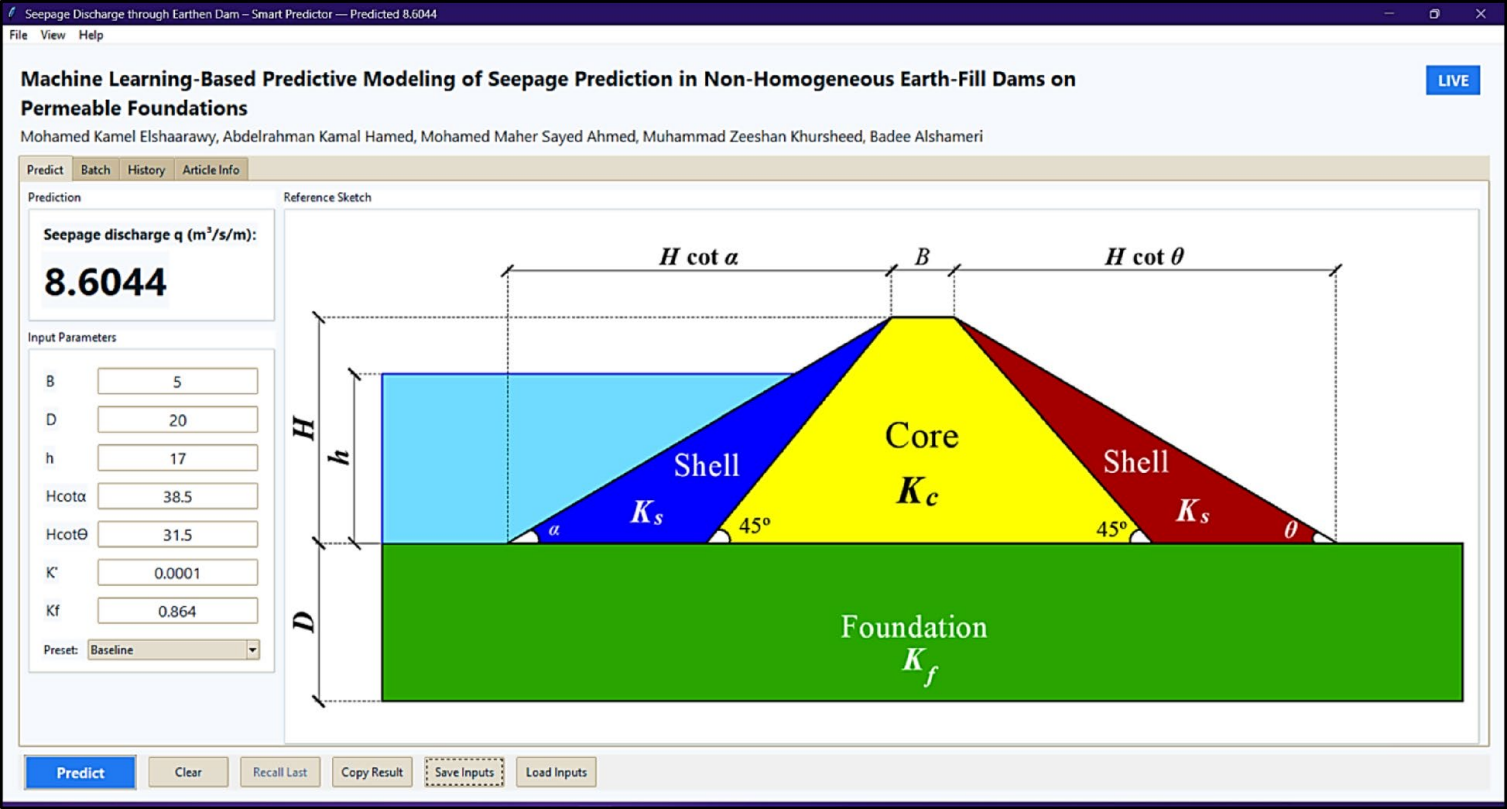
Example screenshot of the desktop-based GUI to predict *q* (m^3^/s/m).

To enable practical use, the best predictive ML model, the CGB model, is deployed through a user-friendly Tkinter-based desktop application^60^, supporting both offline and online use for easy access and instant predictions. Figure 15 presents the standalone desktop graphical user interface (GUI) developed for predicting seepage discharge through non-homogeneous earthfill dams on permeable foundations. The application accepts seven user-defined geometric and hydraulic inputs and instantly returns the predicted seepage discharge (*q*, m³/d/m), with automatic formatting in standard or scientific notation depending on magnitude. A reference schematic is embedded to clarify how each parameter maps to the physical dam profile, while tabs and toolbars allow batch evaluation, history tracking, and input management. The GUI is openly accessible at https://github.com/mkamel24/dam.

From an engineering perspective, the proposed framework can be directly applied in design and safety evaluation by using the developed open-access GUI to rapidly estimate seepage discharge under different hydraulic and geometric conditions. The predicted seepage rates can be readily compared with the design of seepage capacity ($\:{Q}_{\mathrm{design}}$) to support screening-level safety checks and operational decision-making. However, seepage control decisions based on ML predictions are inherently constrained by the governing input parameters, as highlighted by the SHAP-based interpretation. The SHAP analysis clarifies how changes in key variables may positively or negatively influence seepage discharge, indicating which parameters can be effectively controlled through design or remediation measures and which reflect inherent site conditions.

### Models verification

#### Test dataset

**Fig. 16:**
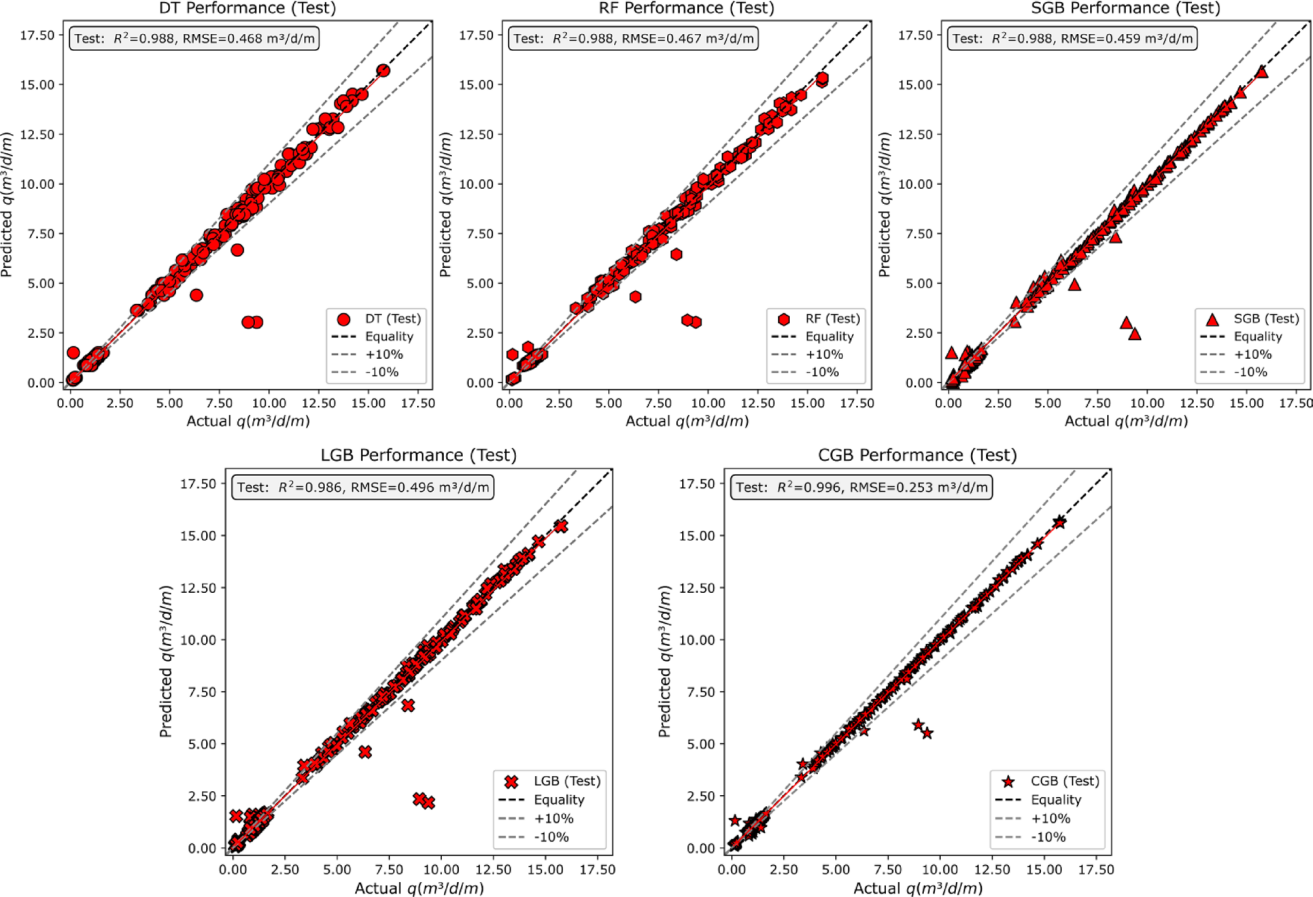
Verification of ML models via scatter plots using the unseen dataset (Testing dataset).

Figure 16 presents scatter plots of predicted versus observed seepage discharge for the testing dataset, enabling a direct comparison of model performance under unseen conditions. Across all models, the data points generally align along the line of equality, indicating good overall agreement between predicted and actual values. The DT and RF models exhibit noticeable dispersion around the equality line, with several points falling outside the ± 10% bounds, particularly at low-to-intermediate discharge values. This behavior is reflected in their comparable coefficients of determination ($\:{R}^{2}=0.988$ for both models) and relatively higher RMSE values (0.468 m³/d/m for DT and 0.467 m³/d/m for RF), indicating similar predictive accuracy and error spread in the testing stage.

In contrast, the SGB and LGB models show a tighter clustering of points around the equality line, with fewer deviations beyond the ± 10% limits across the discharge range. These patterns are accompanied by high $\:{R}^{2}$values (0.988 for SGB and 0.986 for LGB) and slightly lower RMSE values for SGB (0.459 m³/d/m) compared to LGB (0.496 m³/d/m), suggesting differences in dispersion despite similar correlation strength. The CGB model exhibits the most compact distribution of points around the equality line, with minimal scatter and limited outliers, particularly at higher discharge values. This visual behavior is supported by the highest coefficient of determination ($\:{R}^{2}=0.996$) and the lowest RMSE (0.253 m³/d/m) among the tested models. Overall, the comparison demonstrates that while all models maintain strong correlation on the testing dataset, they differ in the degree of dispersion and error magnitude, with boosting-based models generally showing tighter agreement between predicted and observed seepage discharge.

#### Case study: Hub dam - pakistan

**Fig. 17:**
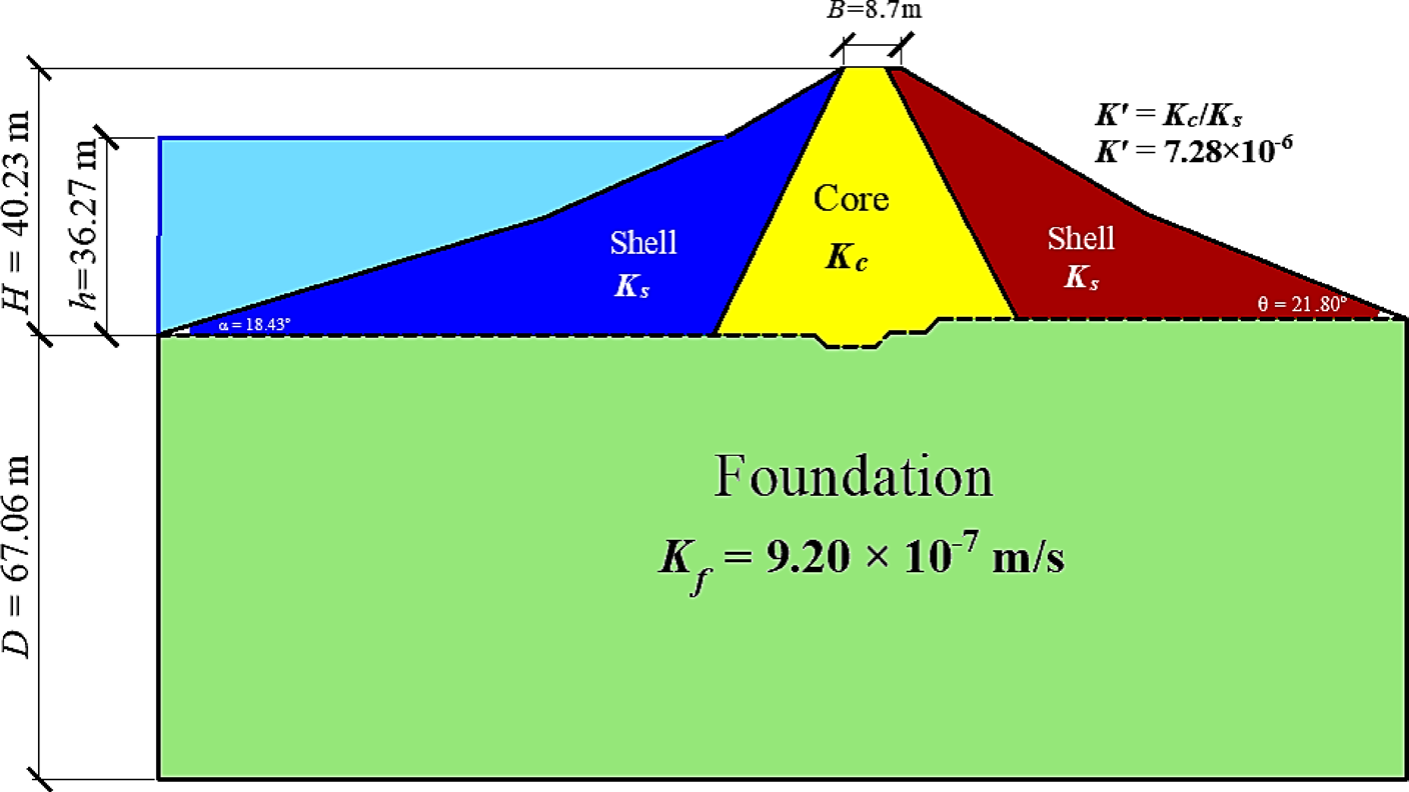
Hub Dam cross-section used in the validation process via the best predictive model (CGB).

To further validate the predictive capability of the proposed CGB model, an independent case study was conducted on Hub Dam, an earthfill dam located approximately 35 km northeast of Karachi, Pakistan (25°15′N, 67°07′E)^28^. Hub Dam has been extensively investigated in previous seepage studies, making it a suitable benchmark for external validation. Figure 17 illustrates the dam cross-section together with the adopted geometric configuration and hydraulic properties of the core, shell, and foundation materials.

Table 5 compares the seepage discharge values predicted by the CGB model, the empirical equation proposed by Khursheed et al.^41^, and the numerical SEEP/W model reported by Arshad and Babar^28^ for three reservoir water levels. The results demonstrate a strong agreement between the CGB predictions and both reference approaches. Across all examined water levels, the CGB model yields seepage discharge estimates that closely match the numerical and empirical results, with percentage differences consistently remaining below 10%. For instance, at a water level of 339 m, the CGB-predicted seepage discharge (4.529 m³/d/m) differs by only 0.81% from the numerical SEEP/W result (4.493 m³/d/m), while the empirical equation predicts 4.225 m³/d/m, corresponding to a larger deviation of approximately 7.2%. In terms of predictive performance, the CGB model achieves a high coefficient of determination during the testing stage (R² = 0.996), which is fully comparable to the numerical modeling efficiency reported by Arshad and Babar^28^ (model efficiency ≈ 99.60%). Moreover, the CGB model outperforms the empirical formulation of Khursheed et al.^41^, which reported a lower coefficient of determination (R² ≈ 0.96) in similar seepage applications. These findings confirm that the CGB model not only reproduces physically based numerical results with high fidelity but also provides improved accuracy over empirical equations, underscoring its reliability and suitability for practical seepage assessment of earthfill dams.

**Table 5:** Verification of the best model in predicting seepage of Hub Dam - Pakistan against previous studies.

Water level	q (m^3^/d/m)		
	Numerical model Arshad and Babar [28]	Empirical equation Khursheed et al. [41]	CGB (Present study)
270	1.762	1.624	1.865
339	4.493	4.225	4.529
346	4.648	4.484	4.614

## Conclusions

This study developed and evaluated Bayesian-optimized machine-learning models for predicting seepage discharge through non-homogeneous earthfill dams on permeable foundations. Seven physically meaningful geometric and hydraulic parameters were used to train and compare five ML algorithms under a comprehensive evaluation framework that included training (80%), validation (10%), and testing (10%) datasets, cross-validation, residual error analysis, rank aggregation, and SHAP-based interpretability. The key conclusions are summarized as follows:

- The CGB model achieved the best overall performance, yielding the lowest RMSE (0.0274 m³/d/m training and 0.186 m³/d/m validation) and consistently high accuracy (R² = 0.9981 in validation stage), with minimal bias and uncertainty.
- Cross-validation results confirmed the superiority of boosting-based models, with CGB attaining the lowest mean RMSE, SGB showing the highest stability, and LGB remaining consistently competitive.
- Goodness-of-fit analysis revealed tight clustering around the 1:1 line for boosting models, particularly CGB and SGB, whereas RF and DT showed larger dispersion and weaker generalization.
- Residual Error Curves (RECs) indicated that CGB, SGB, and LGB concentrated most predictions at low residual errors, while DT and RF exhibited broader error distributions.
- Rank aggregation across seven statistical indices consistently ranked CGB first, followed by SGB and LGB, confirming robustness across multiple performance criteria.
- SHAP analysis identified upstream water head (*h*) and hydraulic conductivity ratio (*K′*) as dominant drivers of seepage, with geometric parameters (*B*, *D*) exerting secondary influence and slope parameters having minor effects.
- A desktop GUI was developed to support real-time prediction, batch evaluation, and result tracking, enabling practical engineering use.
- Independent testing and a Hub Dam case study verified the CGB model’s reliability (R² = 0.996), with prediction differences consistently below 10% relative to numerical and empirical references.

## Limitations and future work

Despite the strong performance of the proposed framework, several limitations should be acknowledged, as follows:

- The ML models were trained primarily on a numerically generated database, which, although physically consistently and widely used in seepage studies, introduces uncertainty associated with FEM-generated ground truths and may not fully represent field conditions affected by construction defects, aging, and in situ heterogeneity.
- The lack of direct field-measured seepage data limits full validation under real operational conditions.
- Although robust generalization was demonstrated using unseen testing data and an independent case study, extrapolation beyond the investigated parameter ranges and dam configurations remains uncertain.
- Uncertainty quantification was mainly represented by the $\:{U}_{95}$metric, which does not fully capture epistemic and aleatory uncertainties.
- The present framework focuses on steady-state seepage, without accounting for transient or time-dependent phenomena.

Future research should integrate field monitoring data, adopt probabilistic uncertainty modeling (e.g., Bayesian or ensemble-based approaches), and extend the methodology to transient and three-dimensional seepage analysis, as well as real-time, sensor-driven prediction, to further enhance applicability in advanced dam safety management.

## Data Availability

Data is available from the corresponding author upon reasonable request.
